# Mobile Phone Call Duration and Cardiovascular Outcomes: Prospective and Genetic Evidence on a Potential Depression-Related Pathway

**DOI:** 10.3390/bioengineering13091035

**Published:** 2026-09-05

**Authors:** Yanxin Yin, Yan Luo, Tong Liu, Qingpeng Zhang

**Affiliations:** 1 Hebei Vocational University of Technology and Engineering, Xingtai 054000, China; yanxinyin2-c@my.cityu.edu.hk; 2 Department of Data Science, City University of Hong Kong, Hong Kong 999077, China; 3 City University of Hong Kong (Dongguan), Dongguan 523808, China; luo.yan@my.cityu.edu.hk; 4 Tianjin Key Laboratory of Ionic-Molecular Function of Cardiovascular Disease, Department of Cardiology, Tianjin Institute of Cardiology, Second Hospital of Tianjin Medical University, Tianjin 300211, China; liutong@tmu.edu.cn; 5 Department of Pharmacology and Pharmacy, LKS Faculty of Medicine, The University of Hong Kong, Hong Kong 999077, China; 6 Musketeers Foundation Institute of Data Science, The University of Hong Kong, Hong Kong 999077, China; 7 The University of Hong Kong-Shenzhen Hospital, Shenzhen 518053, China; 8 Shenzhen Loop Area Institute, Shenzhen 518000, China

**Keywords:** cardiovascular disease, major depressive disorder, mobile phone calls, UK Biobank, Mendelian randomization

## Abstract

Background: Mobile phone use has been associated with cardiovascular disease (CVD), but the role of major depressive disorder (MDD) in this relationship remains uncertain. Methods: We examined self-reported weekly mobile phone call duration and incident heart failure (HF), coronary artery disease (CAD), stroke, and myocardial infarction (MI) in four UK Biobank at-risk cohorts (N = 320,343–328,170) using Cox regression. Baseline MDD was evaluated as a stratification and effect-modification variable. We also conducted two-sample MR and an exploratory two-step MR analysis of the pathway from call duration through MDD to CVD. Genome-wide significant instruments ($p<5\times 10^{-8}$) defined the primary MR analysis; $p<10^{-6}$ was examined as a sensitivity threshold. Results: In fully adjusted Cox models, call duration greater than 6 h/week was associated with CAD (HR 1.18, 95% CI 1.10–1.27) and stroke (HR 1.17, 95% CI 1.05–1.29) but not HF or MI. MDD-by-call-duration interactions were not significant (all interaction p≥0.115). Primary IVW MR estimates supported associations of genetically predicted call duration with MDD (OR 1.56, 95% CI 1.04–2.33) and CAD (OR 1.69, 95% CI 1.13–2.55), whereas estimates for HF, stroke, and MI were imprecise. None of the four primary two-step indirect effects excluded the null, and none remained significant after false-discovery-rate correction at the sensitivity threshold. Conclusions: Longer weekly mobile phone call duration was prospectively associated with CAD and stroke in UK Biobank. The MR findings provide exploratory, but not conclusive, support for an MDD-related pathway.

## 1. Introduction

Globally, cardiovascular diseases (CVDs) are the leading cause of mortality, making the identification of modifiable risk factors a public health priority [1,2]. Mobile phones are used by billions of people worldwide [3], and mobile phone use has therefore been examined as a potential behavioral correlate of cardiovascular health. Proposed explanations include biological responses to radiofrequency exposure and behavioral or psychosocial pathways involving sleep, stress, and sedentary behavior [4,5,6].

Major depressive disorder (MDD) is associated with CVD through biological pathways, including hypothalamic–pituitary–adrenal-axis dysregulation and inflammation, and through behavioral factors such as physical inactivity and poor sleep [7,8,9,10,11]. The relationship between mobile phone use and depression is less clear: mobile communication may facilitate social connection, whereas excessive or problematic use has been associated with depressive symptoms [12,13]. Cross-sectional evidence has suggested that psychological distress may lie on a pathway between phone use and CVD [14], but such designs cannot establish temporal ordering.

We therefore combined two complementary analyses. First, prospective UK Biobank data were used to estimate associations between baseline weekly mobile phone call duration and incident CVD, with baseline MDD treated as a stratification and effect-modification variable. Baseline MDD was not analyzed as a cohort mediator because its onset could precede the measured exposure. Second, two-sample MR was used to examine genetically predicted associations, and two-step MR was used as an exploratory assessment of a potential call-duration–MDD–CVD pathway [15,16]. This design separates prospective phenotypic associations from genetic pathway evidence. The analyses were interpreted as complementary evidence under different assumptions, consistent with triangulation principles, rather than as equivalent demonstrations of mediation [17,18].

## 2. Methods

### 2.1. Two-Sample and Two-Step Mendelian Randomization

MR uses genetic variants as instrumental variables (IVs) and relies on three assumptions: the variants are associated with the exposure, are independent of exposure–outcome confounders, and affect the outcome only through the exposure [17,19]. Two-sample MR estimates variant–exposure and variant–outcome associations in separate GWAS samples [20].

We estimated associations of genetically predicted weekly mobile phone call duration with MDD and each CVD outcome, and associations of genetically predicted MDD with each CVD outcome. For the exploratory two-step analysis, the indirect effect was calculated as the product of the mobile phone call duration–MDD and MDD–CVD coefficients [16].

#### 2.1.1. Data Source

We used publicly available GWAS summary statistics (Table S1). Weekly call duration was derived from UK Biobank (OpenGWAS ID ukb-b-17999; N = 386,626) [21]. The MDD proxy was the FinnGen release 11 ICD-based depression endpoint finngen_R11_F5_DEPRESSIO (53,313 cases and 394,756 controls; total N = 448,069) [22]. CVD outcome associations were obtained from the contributing consortia through IEU OpenGWAS [23,24,25,26]. In the original workflow, phone-use instruments and CVD associations were queried by identifier through the IEU OpenGWAS API, whereas FinnGen depression statistics were read from the downloaded release file. The reproducibility reanalysis used locally archived source files where available and retained allele-harmonized SNP-level snapshots for API-queried sources. The Nanjing University Bioconductor mirror was used only to install R/Bioconductor packages and was not a source of GWAS observations.

#### 2.1.2. Statistical and Sensitivity Analyses

The primary analysis selected SNPs associated with each exposure at genome-wide significance ($p<5\times 10^{-8}$). A sensitivity analysis used the suggestive threshold from the submitted analysis ($p<10^{-6}$). SNPs were clumped at $r^{2}<0.001$ within 10 Mb using a European reference panel. Instrument strength was assessed as $F={\left(\beta /SE\right)}^{2}$; across exposure–outcome analyses, F statistics ranged from 29.92 to 31.20 in the primary analyses and from 24.06 to 24.09 in the sensitivity analyses (Table S3). Analyses used TwoSampleMR version 0.6.13 in R [27]. IVW was designated as the primary estimator, with MR-Egger, weighted median, simple mode, and weighted mode as complementary estimators. Cochran’s Q and I^2^ assessed heterogeneity, the MR-Egger intercept assessed directional pleiotropy, and leave-one-out analyses assessed the influence of individual variants. These instrument-strength and sensitivity diagnostics were reported alongside the primary and sensitivity estimates to support assessment of weak-instrument bias and horizontal pleiotropy [17,28,29].

For two-step MR, product standard errors were calculated with the delta method, and 95% confidence intervals (CIs) were calculated using 1.96 standard errors. Benjamini–Hochberg false-discovery-rate (FDR) correction was applied across the four indirect-effect tests. Proportion estimates and their CIs were considered exploratory because component estimates may not be independent and proportions become unstable when total effects are imprecise.

### 2.2. Cox Proportional Hazards Regression

#### 2.2.1. Study Design and Population

UK Biobank enrolled approximately 500,000 participants aged 37–73 years at 22 assessment centers between 2006 and 2010. Participants completed touchscreen questionnaires, underwent physical measurements, and provided biological samples. Incident outcomes were identified through linkage to hospital and mortality records. UK Biobank received ethics approval, and all participants provided written informed consent [30,31].

**Figure 1:**
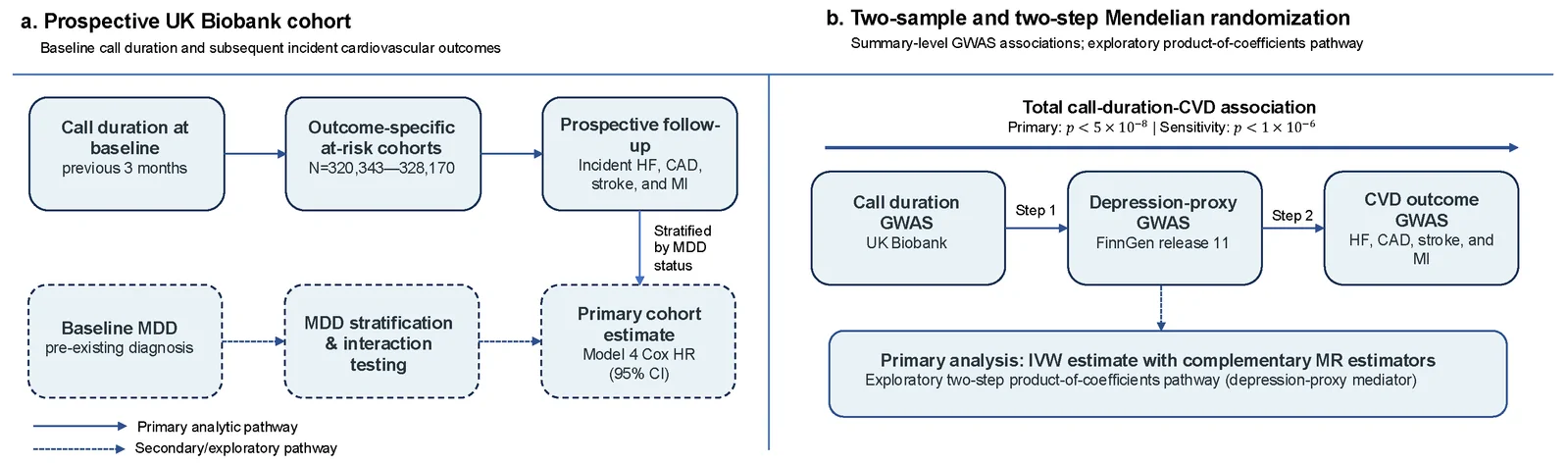
Study design and analytical framework. (**a**) The UK Biobank component estimated associations between baseline weekly mobile phone call duration and incident cardiovascular outcomes in outcome-specific at-risk cohorts. Baseline MDD was used only for stratification and global interaction testing because its temporal order relative to the call-duration measurement was unknown. (**b**) The Mendelian randomization component estimated associations of genetically predicted call duration with MDD and cardiovascular outcomes and of genetically predicted MDD with cardiovascular outcomes. Product-of-coefficients estimates were treated as an exploratory pathway analysis and not as proof of mediation. CAD, coronary artery disease; CI, confidence interval; CVD, cardiovascular disease; HF, heart failure; HR, hazard ratio; IVW, inverse-variance weighted; MDD, major depressive disorder; MI, myocardial infarction.

The overall study design and analytical framework are summarized in Figure 1.

For each outcome, we included participants with complete baseline phone-use and covariate data and excluded those with that CVD at baseline. The resulting at-risk cohorts comprised 328,170 participants for HF, 320,343 for CAD, 324,615 for stroke, and 322,844 for MI.

#### 2.2.2. Measurement of Mobile Phone Use

At baseline, participants reported the average time per week spent making or receiving calls on a mobile phone during the previous 3 months. Response categories were “less than 5 min”, “5–29 min”, “30–59 min”, “1–3 h”, “4–6 h”, and “more than 6 h”. The less-than-5-min category was the reference group.

#### 2.2.3. Measurement of Baseline MDD

Baseline MDD was identified from hospital and mortality records using International Classification of Diseases, 10th Revision (ICD-10) codes F32–F33 (Table S10) [32]. Because recorded MDD could predate the baseline phone-use measurement, it was used for stratification and effect-modification analyses, not as a cohort mediator. This distinction avoids imposing an unsupported exposure–mediator temporal sequence [33,34,35].

#### 2.2.4. Measurement of Covariates

Baseline covariates were obtained from standardized questionnaires and measurements. They included age, sex, height, education, smoking status, alcohol use, diabetes, cancer, and body mass index (BMI; weight in kilograms divided by height in meters squared). Socioeconomic status was assessed with the area-level Townsend deprivation index derived from residential postal codes [36,37] (Table S11).

Sleep pattern was derived from chronotype, sleep duration, insomnia symptoms, snoring, and excessive daytime sleepiness. One point was assigned for each low-risk factor: early chronotype, 7–8 h/day of sleep, never or rarely having insomnia symptoms, no self-reported snoring, and no frequent daytime sleepiness. The resulting score ranged from 0 to 5 [38] (Table S11). We classified scores of 4–5 as healthy, 2–3 as intermediate, and 0–1 as poor.

#### 2.2.5. Assessment of Outcome

The outcomes were incident HF, CAD, MI, and stroke, identified through linkage to hospital admissions and death records. Follow-up ran from baseline assessment until the first occurrence of the outcome, death, loss to follow-up, or the country-specific administrative censoring date (28 February 2018 for Wales and 31 March 2021 for Scotland and England) [36].

Based on ICD-10 codes [32], CAD was defined by I20–I25, HF by I50, MI by I21–I22, and stroke by I60–I64 (Table S9).

#### 2.2.6. Statistical Analysis

Baseline characteristics were summarized as mean ± standard deviation for continuous variables and counts (percentages) for categorical variables [39]. Associations with incident CVD were estimated using Cox proportional hazards models [40]. Model 1 was unadjusted. Model 2 was adjusted for age, sex, and BMI. Model 3 was additionally adjusted for Townsend deprivation index, diabetes, and cancer. Model 4 was additionally adjusted for smoking, alcohol use, sleep pattern, and education. Coded categorical covariates were modeled as factors.

Model 4 was the primary cohort model; Models 1–3 and MDD-stratified estimates were secondary. We did not apply multiplicity correction to category-specific Cox estimates; therefore, secondary and subgroup findings were interpreted cautiously. Fully adjusted analyses were stratified by baseline MDD. Effect modification was evaluated with a 5-degree-of-freedom global Wald test for the interaction between call-duration category and baseline MDD. The proportional-hazards assumption was assessed using scaled Schoenfeld residuals. Where the call-duration term showed evidence of non-proportionality, the HR was interpreted as an association averaged over follow-up. All tests were two-sided. Cohort analyses used time since baseline as the time scale and were fitted in R version 4.4.1 using the survival package and coxph with Efron ties.

## 3. Results

### 3.1. Mendelian Randomization Results

**Figure 2:**
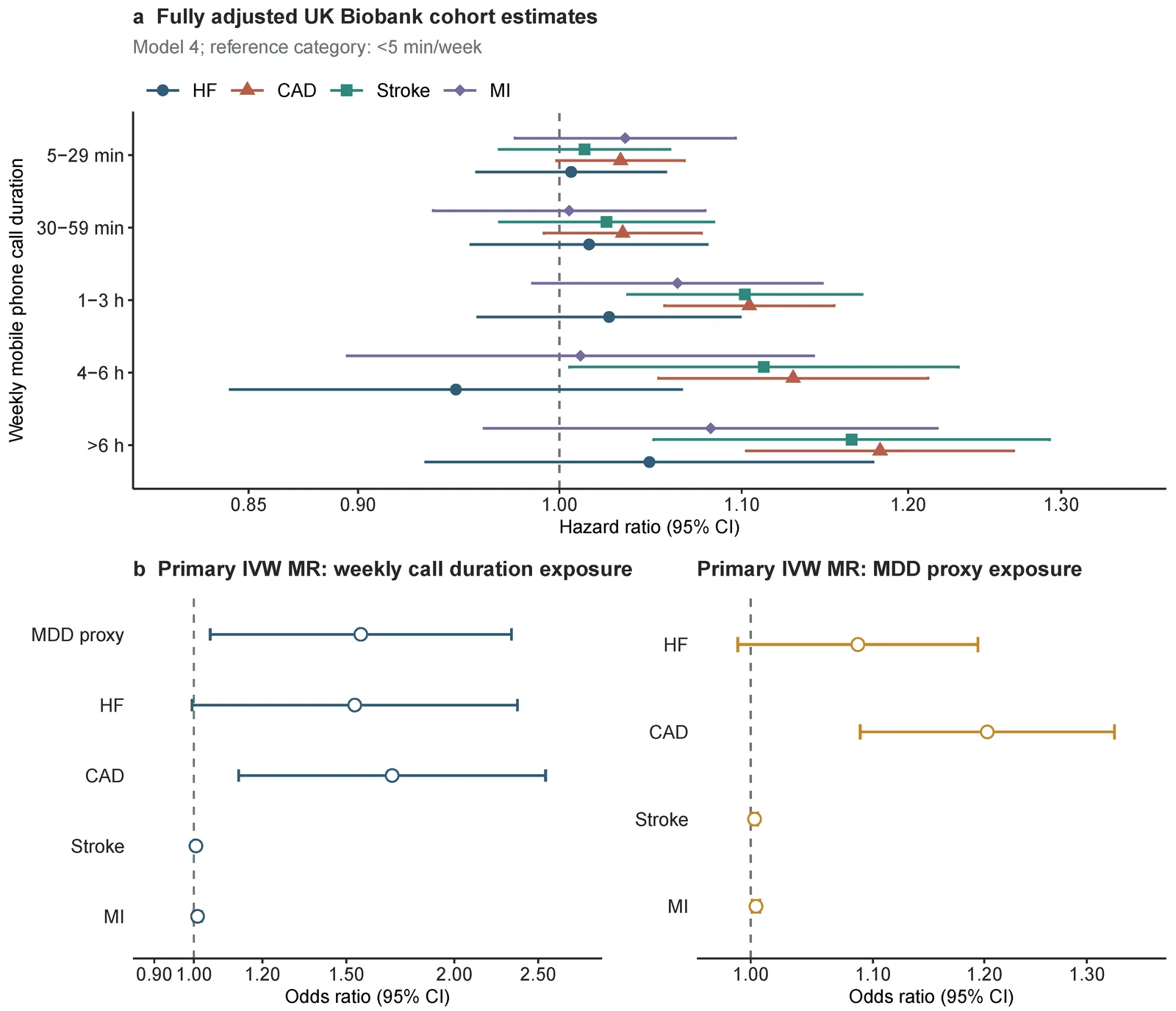
Primary cohort and Mendelian randomization estimates. (**a**) Hazard ratios and 95% confidence intervals for all weekly mobile phone call-duration categories in the fully adjusted Model 4 Cox analyses. The reference category was less than 5 min/week. (**b**) Odds ratios and 95% confidence intervals from the primary inverse-variance weighted Mendelian randomization analyses using genome-wide-significance instruments ($p<5\times 10^{-8}$). The two MR subpanels use different logarithmic x-axis ranges because the effect scales differed substantially by exposure. All designated primary outcomes are shown irrespective of statistical significance. Exact estimates are reported in the corresponding tables. CAD, coronary artery disease; CI, confidence interval; HF, heart failure; HR, hazard ratio; IVW, inverse-variance weighted; MDD, major depressive disorder; MI, myocardial infarction; MR, Mendelian randomization; OR, odds ratio.

In the primary genome-wide-significance analysis, genetically predicted weekly mobile phone call duration was associated with MDD (IVW OR 1.56, 95% CI 1.04–2.33; p=0.030) and CAD (OR 1.69, 95% CI 1.13–2.55; p=0.011) (Table 1; Figure 2b). Estimates for HF (OR 1.53, 95% CI 1.00–2.37; p=0.053), stroke (OR 1.006, 95% CI 0.997–1.014; p=0.179), and MI (OR 1.010, 95% CI 0.999–1.021; p=0.072) were imprecise. Genetically predicted MDD was associated with CAD, stroke, and MI but not HF. Complementary estimators were directionally consistent for the call-duration–CAD association, although several other associations were not reproduced by MR-Egger or mode-based estimators (Table S2).

Cochran’s Q indicated heterogeneity for call duration with MDD, HF, and CAD (Table S3). The MR-Egger intercept did not indicate directional pleiotropy for most primary analyses, but the call-duration–stroke analysis had an intercept p=0.043. Leave-one-out estimates did not reverse direction, but these diagnostics and the limited number of genome-wide significant call-duration instruments reduce confidence in broad causal interpretation.

None of the four primary indirect-effect CIs excluded zero (Table 2). At the $p<10^{-6}$ sensitivity threshold, indirect effects were nominally significant for HF, CAD, and stroke, but none remained significant after FDR correction (BH-FDR p=0.054, 0.054, 0.061, and 0.077 for HF, CAD, stroke, and MI, respectively). Accordingly, these estimates are treated as exploratory pathway evidence rather than confirmed mediation.

### 3.2. UK Biobank Cohort Results

The CAD analytic cohort included 320,343 participants; the outcome-specific cohorts ranged from 320,343 to 328,170 participants. Participants reporting longer call duration were younger, more often male, and had higher BMI and greater socioeconomic deprivation (Table 3). Baseline MDD prevalence varied only modestly across call-duration categories (7.7–8.6%).

In fully adjusted models, call duration of 1–3 h/week, 4–6 h/week, and more than 6 h/week was associated with CAD (HRs 1.10, 1.13, and 1.18, respectively) and stroke (HRs 1.10, 1.11, and 1.17, respectively) compared with less than 5 min/week (Table 4; Figure 2a). Fully adjusted associations with HF and MI were not statistically significant. Global interaction tests provided no evidence that baseline MDD modified the call-duration association for HF (p=0.115), CAD (p=0.591), stroke (p=0.519), or MI (p=0.778). Stratified estimates are therefore descriptive and are reported in Tables S5–S8.

Scaled Schoenfeld residual tests for the phone-use term were non-significant for HF (p=0.165), CAD (p=0.085), and MI (p=0.569) but suggested non-proportionality for stroke (p=0.021) (Table S4). The stroke HRs are consequently interpreted as associations averaged over follow-up. Global tests were significant because several adjustment covariates also showed time-varying associations.

## 4. Discussion

In this large UK Biobank analysis, longer weekly mobile phone call duration was prospectively associated with incident CAD and stroke after multivariable adjustment, whereas fully adjusted associations with HF and MI were not statistically significant. Baseline MDD did not materially modify these associations. These findings concern time spent making or receiving calls and should not be generalized to total screen time or problematic smartphone use.

The primary MR analysis supported associations of genetically predicted call duration with MDD and CAD but not with HF, stroke, or MI. Results also varied across complementary estimators and showed heterogeneity for several comparisons. Most importantly, none of the primary two-step indirect effects excluded the null. The suggestive-threshold analysis produced nominal indirect associations for HF, CAD, and stroke, but none survived correction for four outcomes. The MR component therefore suggests a possible MDD-related pathway but does not establish mediation.

An MDD-related pathway remains biologically plausible because depression is associated with inflammation, hypothalamic–pituitary–adrenal-axis dysregulation, and adverse health behaviors [41,42,43]. Other correlates of prolonged phone use, including sedentary behavior and sleep disruption, may also contribute [5,6]. However, biological plausibility cannot resolve residual confounding in the cohort analysis or horizontal pleiotropy in MR. In keeping with triangulation principles, agreement or disagreement between the cohort and MR components was therefore treated as evidence to be interpreted under their distinct sources of bias, not as automatic confirmation of a common causal pathway [17,18].

Several limitations are important. Phone call duration was self-reported once at baseline and may not represent later use or other dimensions of digital behavior. Baseline MDD could precede phone exposure, so incident or time-updated MDD was unavailable and cohort mediation was not identifiable. The observational estimates remain susceptible to residual confounding and healthy-user selection. The global proportional-hazards tests were significant because several covariates had time-varying associations, and the stroke-exposure term also showed modest non-proportionality. For MR, the call-duration GWAS was based on UK Biobank, creating possible participant overlap with the cohort analysis and with UK Biobank-derived outcome GWAS; the FinnGen endpoint was a broad depression phenotype used as an MDD proxy; the number of genome-wide significant instruments was limited; several IVW estimates were not supported by MR-Egger or mode-based estimators; and pleiotropy cannot be excluded [16,19]. Finally, indirect-effect proportions rely on scale and independence assumptions and should not be interpreted as precise mechanistic fractions.

## 5. Conclusions

Longer weekly mobile phone call duration was associated with higher risks of CAD and stroke in UK Biobank but not with HF or MI after full adjustment. Primary MR supported call-duration associations with MDD and CAD, while the two-step indirect effects provided exploratory but not conclusive support for an MDD-related pathway. These results justify further longitudinal research using objective, repeated phone-use measures and incident MDD to better characterize this potential pathway.

## Figures and Tables

**Table 1:** Primary inverse-variance weighted Mendelian randomization estimates using genome-wide significant instruments ($p<5\times 10^{-8}$).

Exposure	Outcome	SNPs	β (SE)	OR (95% CI)	*p* Value
Weekly mobile phone call duration	Major depressive disorder	11	0.4442 (0.2045)	1.559 (1.044–2.328)	0.030
Weekly mobile phone call duration	Heart failure	9	0.4281 (0.2209)	1.534 (0.995–2.365)	0.053
Weekly mobile phone call duration	Coronary artery disease	9	0.5276 (0.2083)	1.695 (1.127–2.550)	0.011
Weekly mobile phone call duration	Stroke	11	0.0055 (0.0041)	1.006 (0.997–1.014)	0.179
Weekly mobile phone call duration	Myocardial infarction	11	0.0098 (0.0054)	1.010 (0.999–1.021)	0.072
Major depressive disorder	Heart failure	20	0.0836 (0.0478)	1.087 (0.990–1.194)	0.080
Major depressive disorder	Coronary artery disease	19	0.1846 (0.0506)	1.203 (1.089–1.328)	<0.001
Major depressive disorder	Stroke	24	0.0030 (0.0013)	1.003 (1.001–1.005)	0.018
Major depressive disorder	Myocardial infarction	24	0.0041 (0.0016)	1.004 (1.001–1.007)	0.008

CI, confidence interval; MDD, major depressive disorder; OR, odds ratio. Estimates are expressed per unit increase in the genetically predicted exposure as scaled in the source GWAS.

**Table 2:** Exploratory two-step MR estimates for the pathway from mobile phone call duration through MDD to cardiovascular outcomes.

Instrument Threshold	Outcome	Indirect β (95% CI)	*p* Value	BH-FDR P	Proportion	Proportion 95% CI
$p<5\times 10^{-8}$	HF	0.0372 (−0.0163–0.0906)	0.173	0.173	8.7%	−6.6–23.9%
$p<5\times 10^{-8}$	CAD	0.0820 (−0.0041–0.1681)	0.062	0.145	15.5%	−4.7–35.8%
$p<5\times 10^{-8}$	STROKE	0.0013 (−0.0003–0.0029)	0.109	0.145	24.0%	−21.7–69.8%
$p<5\times 10^{-8}$	MI	0.0018 (−0.0003–0.0040)	0.094	0.145	18.7%	−11.2–48.5%
$p<10^{-6}$	HF	0.0291 (0.0034–0.0549)	0.027	0.054	8.7%	−0.7–18.2%
$p<10^{-6}$	CAD	0.0359 (0.0053–0.0664)	0.021	0.054	7.7%	0.4–15.0%
$p<10^{-6}$	STROKE	0.0007 (0.0000–0.0013)	0.046	0.061	11.6%	−3.1–26.3%
$p<10^{-6}$	MI	0.0008 (−0.0001–0.0017)	0.077	0.077	7.7%	−2.3–17.6%

The indirect effect is the product of the IVW estimates for mobile phone call duration → MDD and MDD → outcome. Product standard errors use the delta method. Proportion confidence intervals assume independence among component estimates and are therefore exploratory. BH-FDR, Benjamini–Hochberg false-discovery-rate correction.

**Table 3:** Baseline characteristics of the CAD-free UK Biobank cohort by weekly mobile phone call duration.

Characteristic	<5 min	5–29 min	30–59 min	1–3 h	4–6 h	>6 h	p Value
Number of participants	64,923	125,256	55,964	46,461	13,630	14,109	
Age, years (mean (SD))	58.4 (7.6)	56.5 (7.9)	54.7 (7.8)	52.9 (7.7)	51.5 (7.4)	50.4 (7.0)	<0.001
Sex, female (%)	37,501 (57.8)	74,741 (59.7)	30,984 (55.4)	23,309 (50.2)	6361 (46.7)	6096 (43.2)	<0.001
Townsend deprivation index (mean (SD))	−1.8 (2.8)	−1.6 (2.9)	−1.3 (3.1)	−1.2 (3.1)	−1.1 (3.2)	−1.1 (3.3)	<0.001
BMI, kg/m^2^ (mean (SD))	27.0 (4.6)	27.2 (4.7)	27.5 (4.7)	27.7 (4.8)	28.1 (4.9)	28.4 (4.9)	<0.001
Height, cm (mean (SD))	168.0 (9.1)	167.9 (9.2)	168.7 (9.3)	169.7 (9.4)	170.2 (9.5)	170.8 (9.4)	<0.001
Diabetes, *n* (%)	3007 (4.6)	5260 (4.2)	2458 (4.4)	1926 (4.1)	543 (4.0)	603 (4.3)	<0.001
Cancer, *n* (%)	5579 (8.6)	9787 (7.8)	3948 (7.1)	2940 (6.3)	729 (5.3)	737 (5.2)	<0.001
University education, *n* (%)	21,197 (32.6)	42,698 (34.1)	19,361 (34.6)	15,949 (34.3)	4479 (32.9)	4340 (30.8)	<0.001
Smoking, *n* (%)	5157 (7.9)	11,427 (9.1)	6210 (11.1)	6093 (13.1)	1973 (14.5)	2410 (17.1)	<0.001
Drinking, *n* (%)	60,176 (92.7)	117,322 (93.7)	52,495 (93.8)	43,460 (93.5)	12,714 (93.3)	13,036 (92.4)	<0.001
Sleep pattern, *n* (%)							<0.001
Healthy	25,633 (39.5)	48,135 (38.4)	20,712 (37.0)	16,360 (35.2)	4606 (33.8)	4512 (32.0)	
Intermediate	36,786 (56.7)	72,061 (57.5)	32,745 (58.5)	27,842 (59.9)	8258 (60.6)	8701 (61.7)	
Poor	2504 (3.9)	5060 (4.0)	2507 (4.5)	2259 (4.9)	766 (5.6)	896 (6.4)	
Baseline MDD, *n* (%)	4972 (7.7)	10,101 (8.1)	4791 (8.6)	3925 (8.4)	1101 (8.1)	1125 (8.0)	<0.001

BMI, body mass index; CAD, coronary artery disease; MDD, major depressive disorder.

**Table 4:** Associations between weekly mobile phone call duration and incident cardiovascular outcomes.

Weekly Call Duration	Cases/N	Model 1 HR (95% CI), *p*	Model 2 HR (95% CI), *p*	Model 3 HR (95% CI), *p*	Model 4 HR (95% CI), *p*
**HF**; global MDD interaction *p* = 0.115					
<5 min	2545/66,873	Reference	Reference	Reference	Reference
5–29 min	4202/128,369	0.85 (0.81–0.90), <0.001	1.01 (0.96–1.06), 0.694	1.00 (0.96–1.05), 0.893	1.01 (0.96–1.06), 0.808
30–59 min	1720/57,265	0.78 (0.73–0.83), <0.001	1.05 (0.99–1.12), 0.106	1.02 (0.96–1.09), 0.436	1.02 (0.95–1.08), 0.623
1–3 h	1277/47,399	0.69 (0.65–0.74), <0.001	1.08 (1.00–1.15), 0.036	1.05 (0.98–1.12), 0.205	1.03 (0.96–1.10), 0.458
4–6 h	318/13,897	0.59 (0.52–0.66), <0.001	1.02 (0.90–1.15), 0.770	0.98 (0.87–1.10), 0.741	0.95 (0.84–1.07), 0.368
>6 h	331/14,367	0.59 (0.53–0.66), <0.001	1.14 (1.01–1.28), 0.031	1.10 (0.98–1.24), 0.098	1.05 (0.93–1.18), 0.430
**CAD**; global MDD interaction *p* = 0.591					
<5 min	5460/64,923	Reference	Reference	Reference	Reference
5–29 min	9531/125,256	0.90 (0.87–0.93), <0.001	1.03 (1.00–1.07), 0.047	1.03 (1.00–1.07), 0.073	1.03 (1.00–1.07), 0.061
30–59 min	4012/55,964	0.84 (0.81–0.88), <0.001	1.06 (1.01–1.10), 0.008	1.04 (1.00–1.09), 0.047	1.03 (0.99–1.08), 0.116
1–3 h	3306/46,461	0.83 (0.80–0.87), <0.001	1.14 (1.09–1.19), <0.001	1.12 (1.08–1.17), <0.001	1.10 (1.06–1.15), <0.001
4–6 h	937/13,630	0.80 (0.75–0.86), <0.001	1.18 (1.10–1.27), <0.001	1.17 (1.09–1.25), <0.001	1.13 (1.05–1.21), <0.001
>6 h	977/14,109	0.81 (0.75–0.86), <0.001	1.26 (1.17–1.35), <0.001	1.24 (1.16–1.33), <0.001	1.18 (1.10–1.27), <0.001
**Stroke**; global MDD interaction *p* = 0.519					
<5 min	3097/65,945	Reference	Reference	Reference	Reference
5–29 min	5189/127,042	0.86 (0.83–0.90), <0.001	1.02 (0.97–1.07), 0.407	1.01 (0.97–1.06), 0.612	1.01 (0.97–1.06), 0.566
30–59 min	2090/56,676	0.78 (0.73–0.82), <0.001	1.06 (1.00–1.12), 0.052	1.03 (0.98–1.09), 0.264	1.02 (0.97–1.08), 0.389
1–3 h	1631/46,969	0.73 (0.68–0.77), <0.001	1.15 (1.08–1.22), <0.001	1.12 (1.05–1.19), <0.001	1.10 (1.04–1.17), 0.002
4–6 h	436/13,766	0.66 (0.60–0.73), <0.001	1.19 (1.07–1.31), 0.001	1.15 (1.04–1.27), 0.007	1.11 (1.01–1.23), 0.039
>6 h	426/14,217	0.62 (0.56–0.69), <0.001	1.26 (1.14–1.40), <0.001	1.22 (1.10–1.35), <0.001	1.17 (1.05–1.29), 0.004
**MI**; global MDD interaction *p* = 0.778					
<5 min	1823/65,575	Reference	Reference	Reference	Reference
5–29 min	3240/126,269	0.92 (0.87–0.97), 0.003	1.04 (0.98–1.10), 0.214	1.03 (0.98–1.10), 0.251	1.03 (0.98–1.10), 0.242
30–59 min	1365/56,419	0.86 (0.80–0.93), <0.001	1.03 (0.96–1.11), 0.425	1.02 (0.95–1.09), 0.638	1.01 (0.94–1.08), 0.888
1–3 h	1153/46,701	0.88 (0.81–0.94), <0.001	1.10 (1.02–1.19), 0.010	1.09 (1.01–1.18), 0.023	1.06 (0.99–1.15), 0.110
4–6 h	313/13,696	0.81 (0.72–0.91), <0.001	1.06 (0.94–1.20), 0.329	1.05 (0.93–1.19), 0.405	1.01 (0.90–1.14), 0.859
>6 h	344/14,184	0.86 (0.76–0.96), 0.009	1.17 (1.04–1.32), 0.010	1.16 (1.03–1.30), 0.015	1.08 (0.96–1.22), 0.191

Model 1 was unadjusted. Model 2 adjusted for age, sex, and BMI. Model 3 additionally adjusted for Townsend deprivation index, diabetes, and cancer. Model 4 additionally adjusted for smoking, alcohol use, sleep pattern, and education. Interaction *p* values are 5-df global Wald tests for call-duration category by baseline MDD.

## Data Availability

Individual-level UK Biobank data are available to approved researchers through the UK Biobank access procedure (application 79146). The authors are not permitted to redistribute these individual-level data, which remain subject to the UK Biobank access terms. GWAS summary statistics are publicly available from FinnGen release 11, the IEU OpenGWAS resource, and the GWAS Catalog under the identifiers listed in Table S1 and remain subject to the respective repositories’ terms of use. Downloaded source files were retained in the local analysis archive where available; API-queried sources are represented in the revision analysis by retained allele-harmonized SNP-level snapshots. The revision scripts, figure-generation script, figure source data, and shareable SNP-level inputs are provided in the accompanying code archive and are available at GitHub (https://github.com/LynnYinU/mobile-phone-mdd-cvd-reproducibility; accessed on 25 August 2026) under a CC0 license. The Nanjing University Bioconductor mirror was used to install software packages and did not provide participant or GWAS observations.

## Supplementary Materials

The following supporting information can be downloaded at https://www.mdpi.com/article/10.3390/bioengineering13091035/s1, Figures S1–S36 and Tables S1–S11.
