# Supplementary material for: Mobile Phone Call Duration and Cardiovascular Outcomes: Prospective and Genetic Evidence on a Potential Depression-Related Pathway
Source: Bioengineering (Basel). 2026 Sep 5;13(9):1035. doi: 10.3390/bioengineering13091035 (PMC13603969; doi:10.3390/bioengineering13091035)
Supplement: Supplementary file 1 [file bioengineering-13-01035-s001.zip › bioengineering-4463532-supplementary_materials.pdf]

Article

Supplementary Materials

Yanxin Yin, Yan Luo, Tong Liu and Qingpeng Zhang

Supplementary Materials

Supplementary Figures

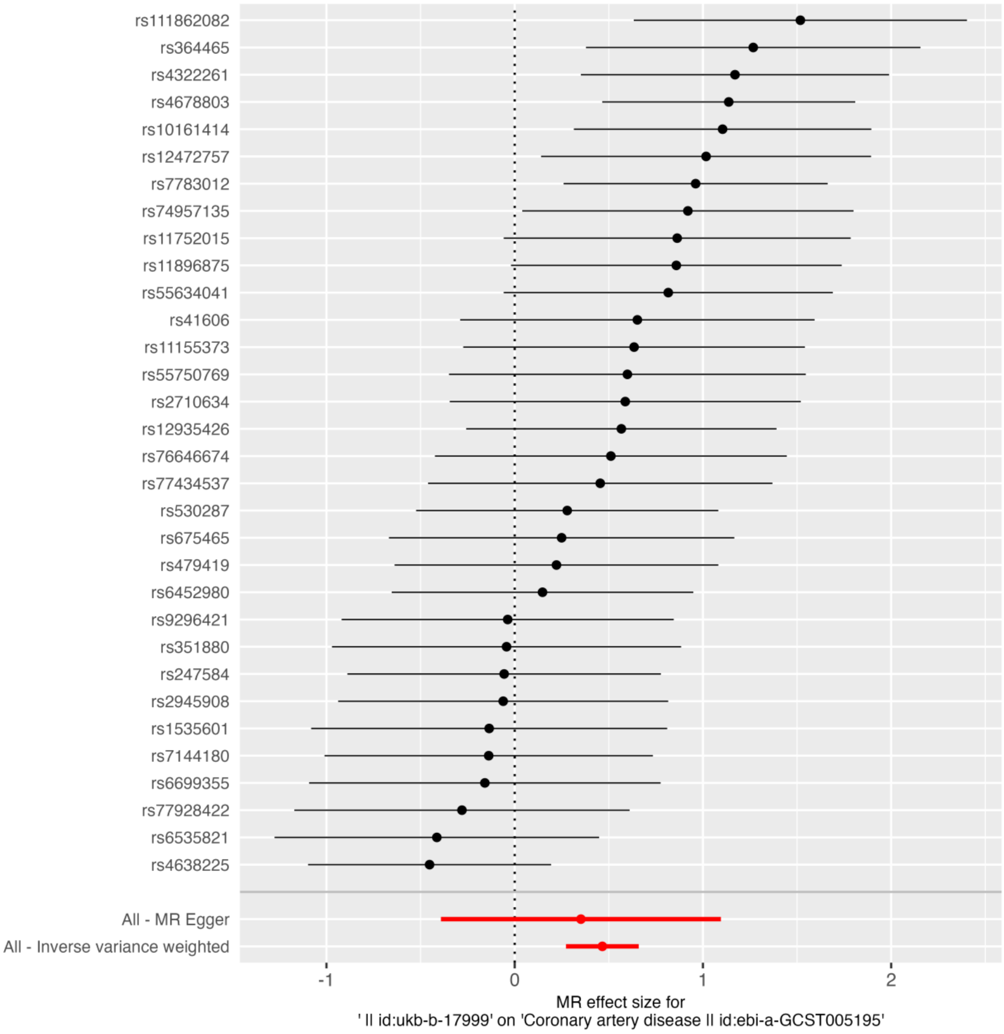

Figure S1. MR sensitivity analysis ($p < 10^{-6}$) of weekly mobile phone call duration on coronary artery disease. (a) Forest plot showing effect estimates of individual SNPs.

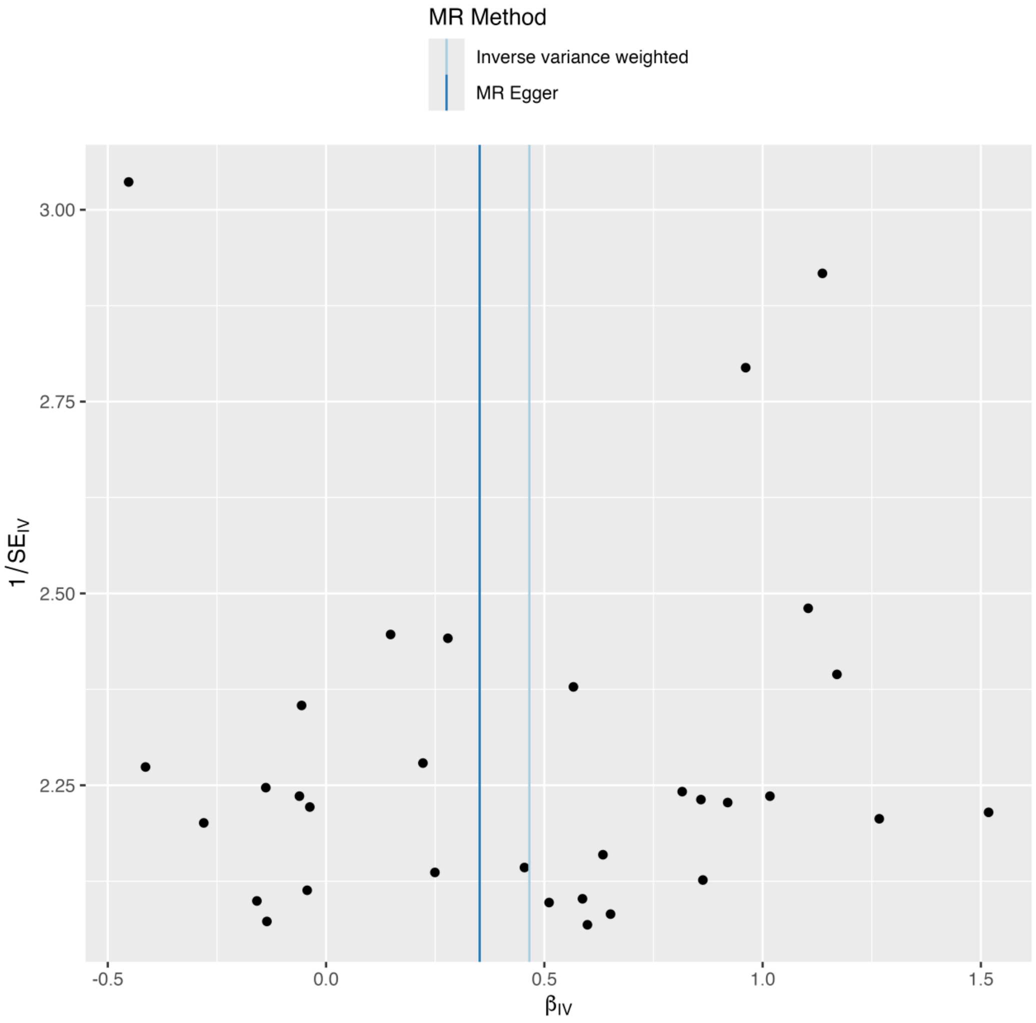

Figure S2. MR sensitivity analysis ($p < 10^{-6}$) of weekly mobile phone call duration on coronary artery disease. (b) Funnel plot assessing potential horizontal pleiotropy.

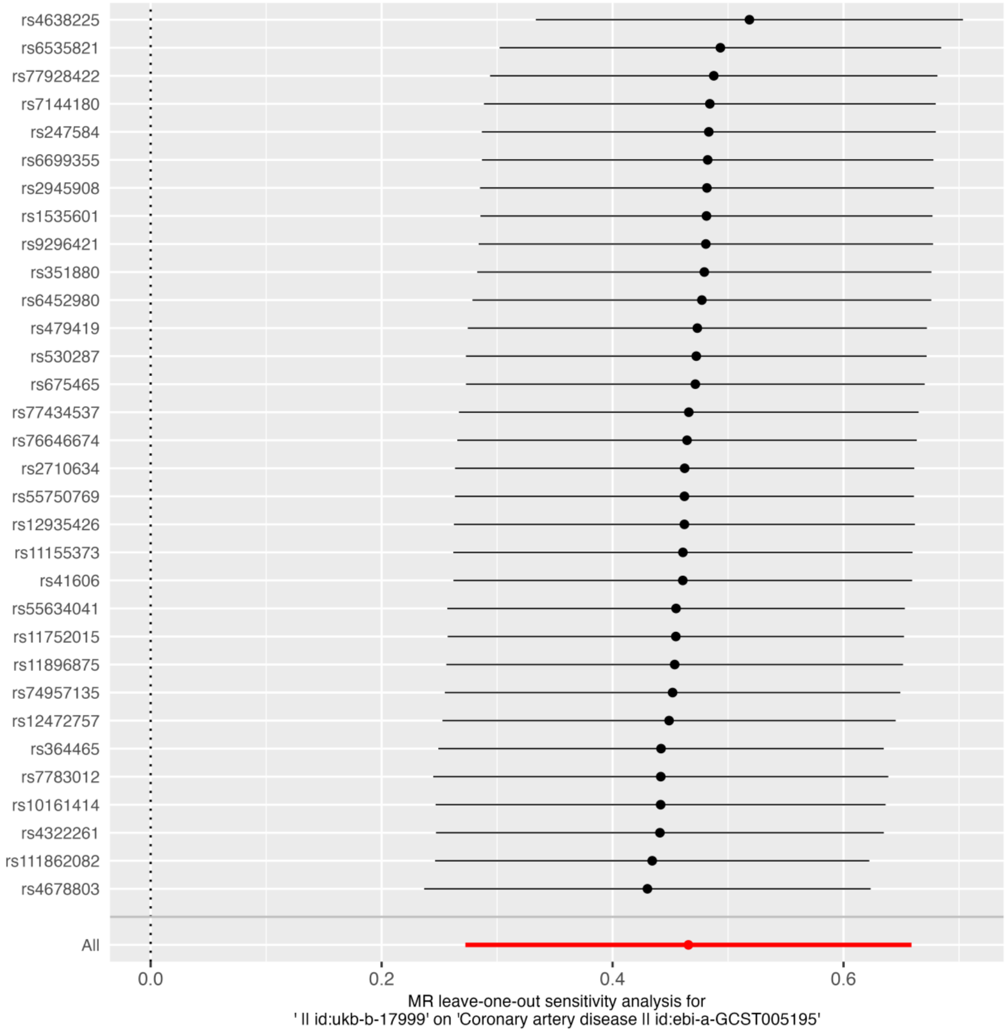

Figure S3. MR sensitivity analysis ($p < 10^{-6}$) of weekly mobile phone call duration on coronary artery disease. (c) Leave-one-out analysis to assess the influence of single SNPs on overall estimates.

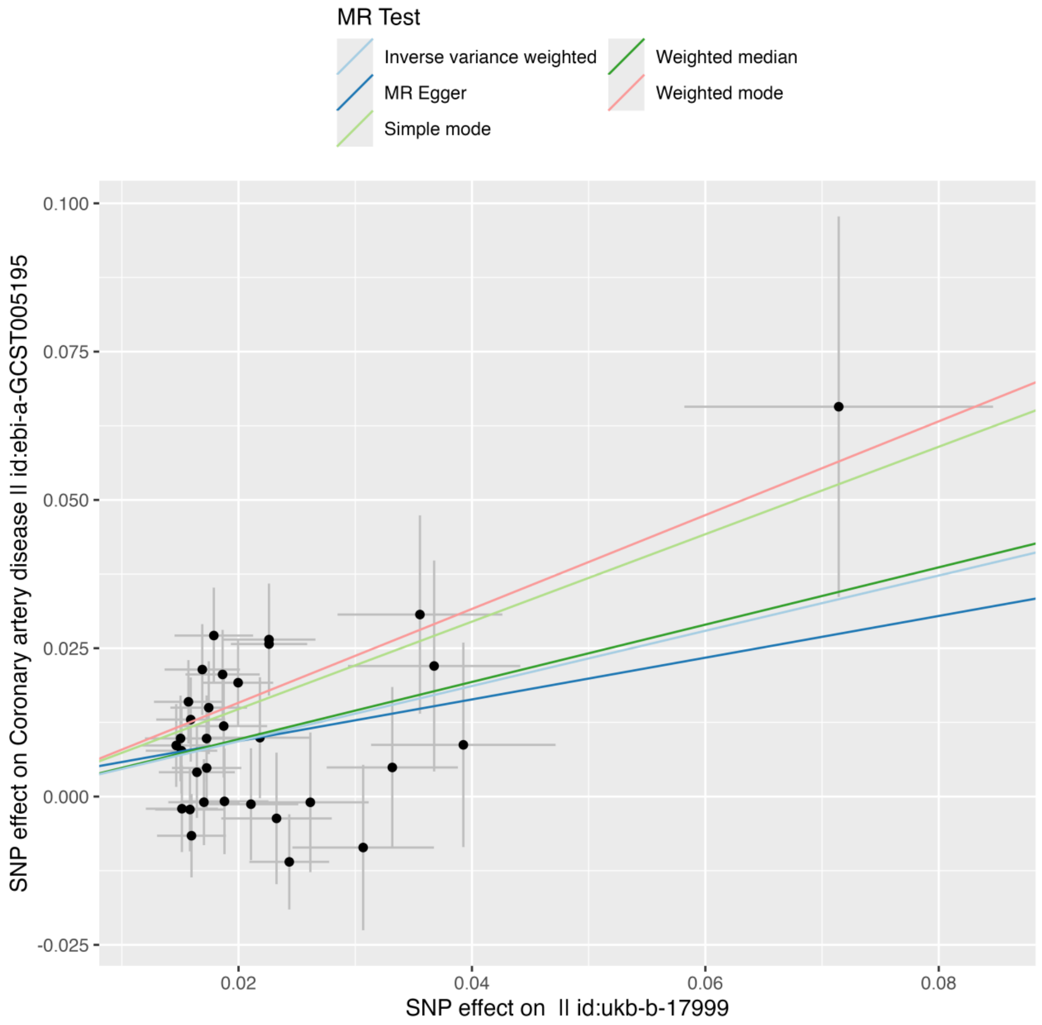

Figure S4. MR sensitivity analysis ($p < 10^{-6}$) of weekly mobile phone call duration on coronary artery disease. (d) Scatter plot of SNP effects on exposure and outcome with MR regression lines.

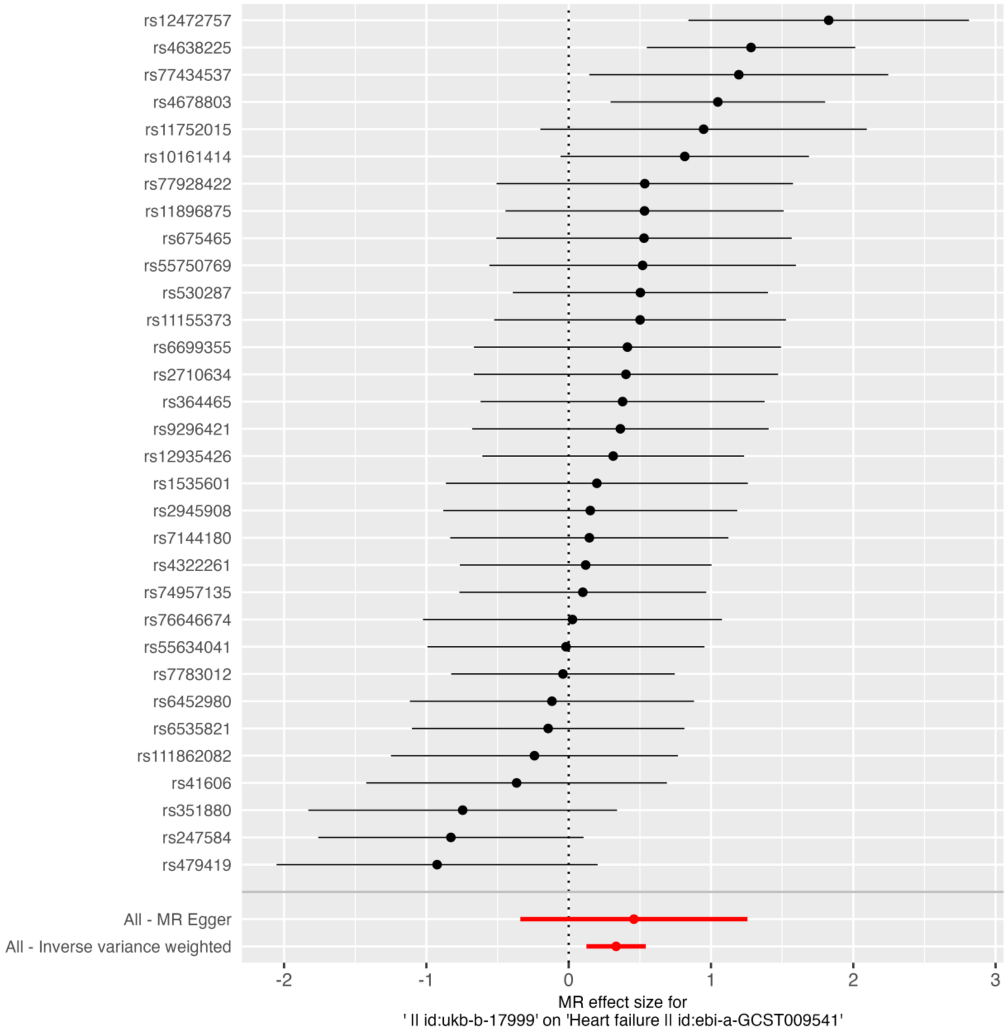

Figure S5. MR sensitivity analysis ($p < 10^{-6}$) of weekly mobile phone call duration on heart failure. (a) Forest plot showing effect estimates of individual SNPs.

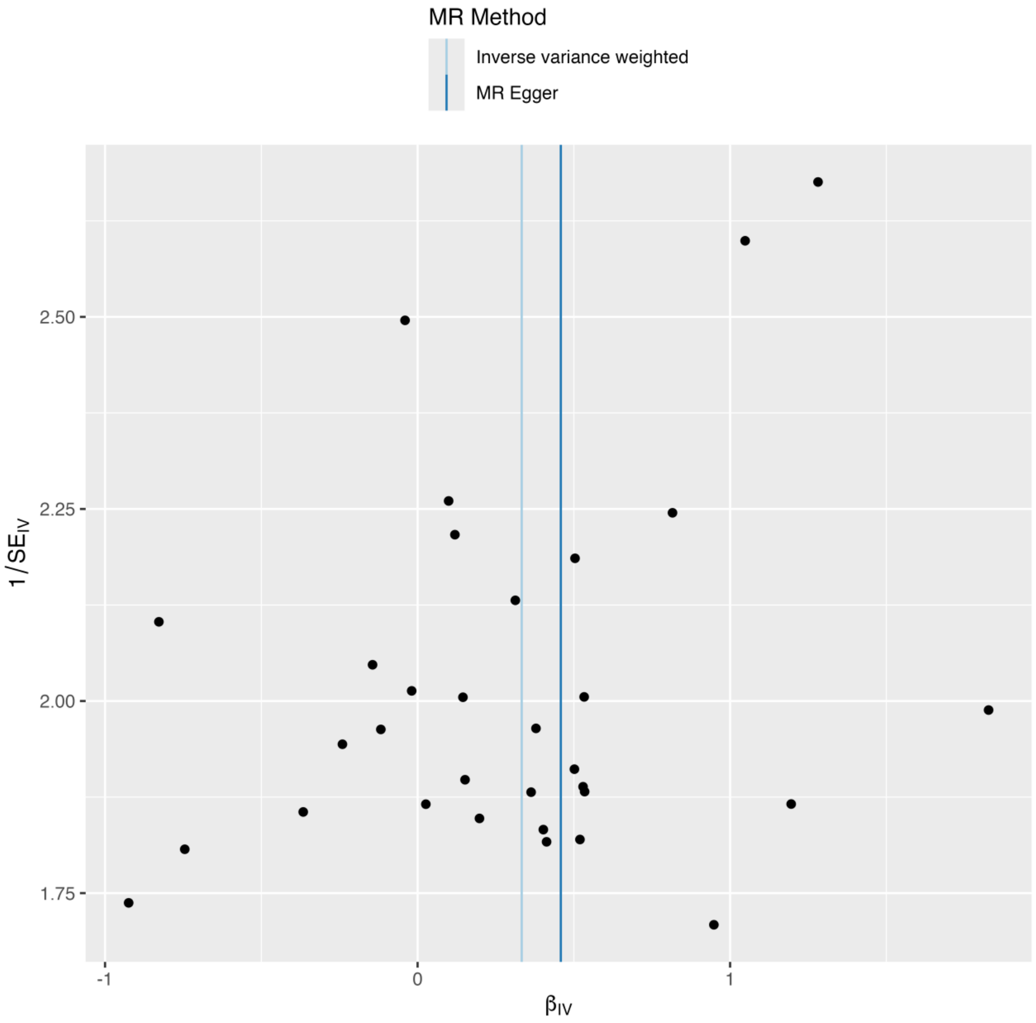

Figure S6. MR sensitivity analysis ($p < 10^{-6}$) of weekly mobile phone call duration on heart failure. (b) Funnel plot assessing potential horizontal pleiotropy.

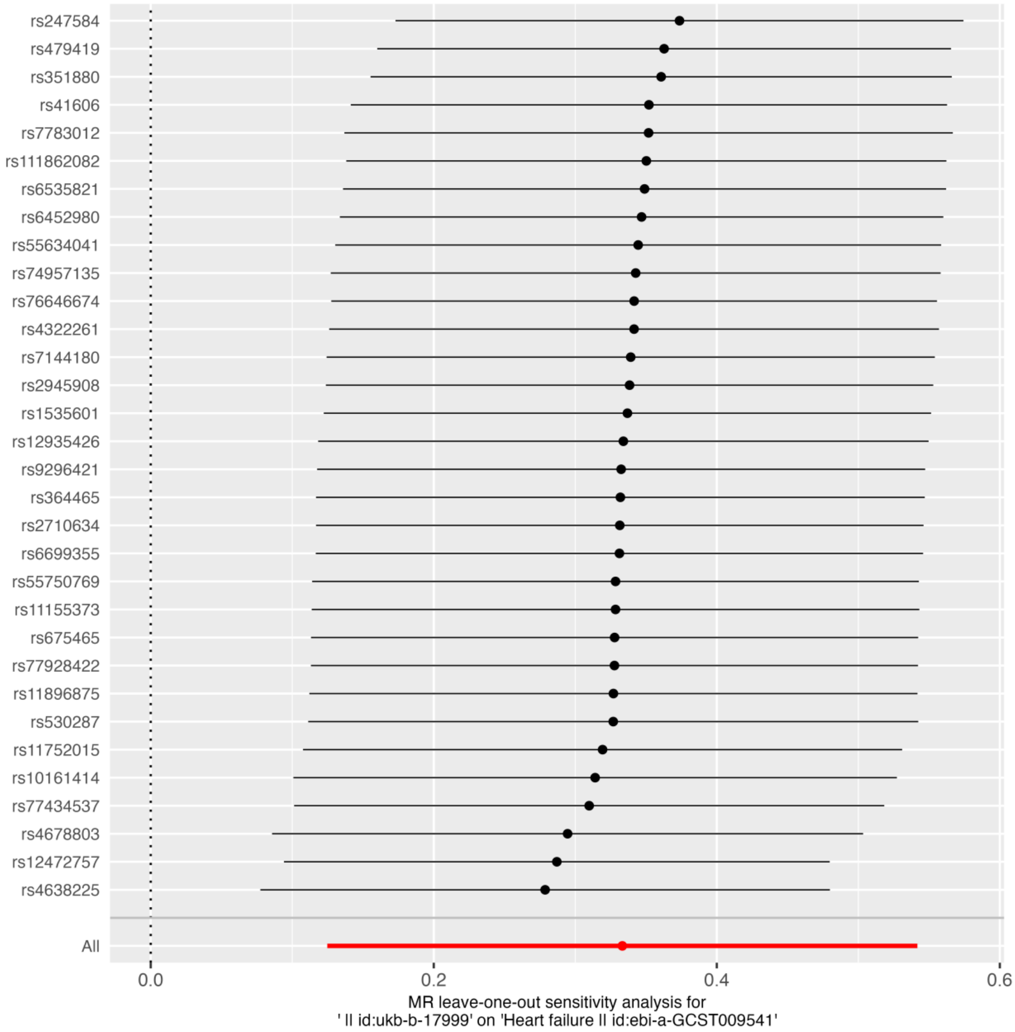

Figure S7. MR sensitivity analysis ($p < 10^{-6}$) of weekly mobile phone call duration on heart failure. (c) Leave-one-out analysis to assess the influence of single SNPs on overall estimates.

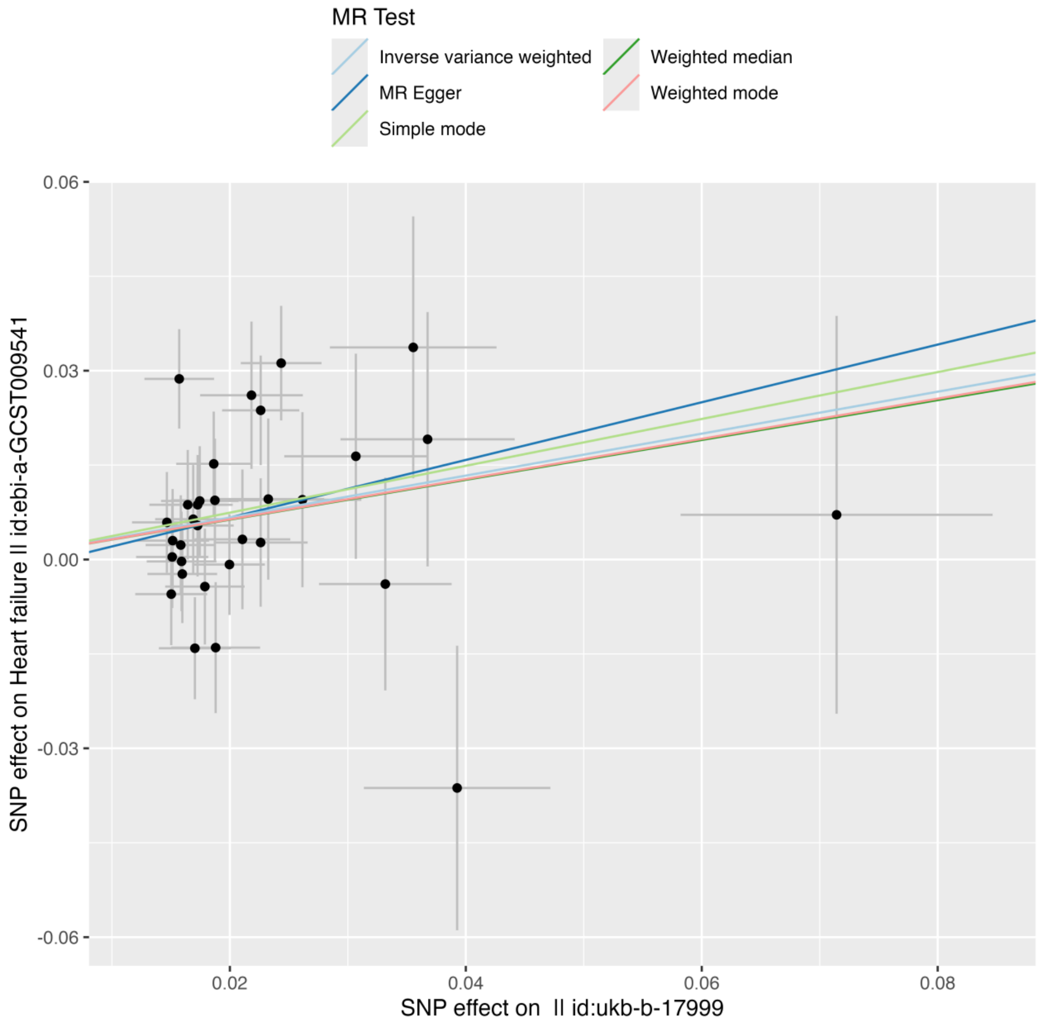

Figure S8. MR sensitivity analysis ($p < 10^{-6}$) of weekly mobile phone call duration on heart failure. (d) Scatter plot of SNP effects on exposure and outcome with MR regression lines.

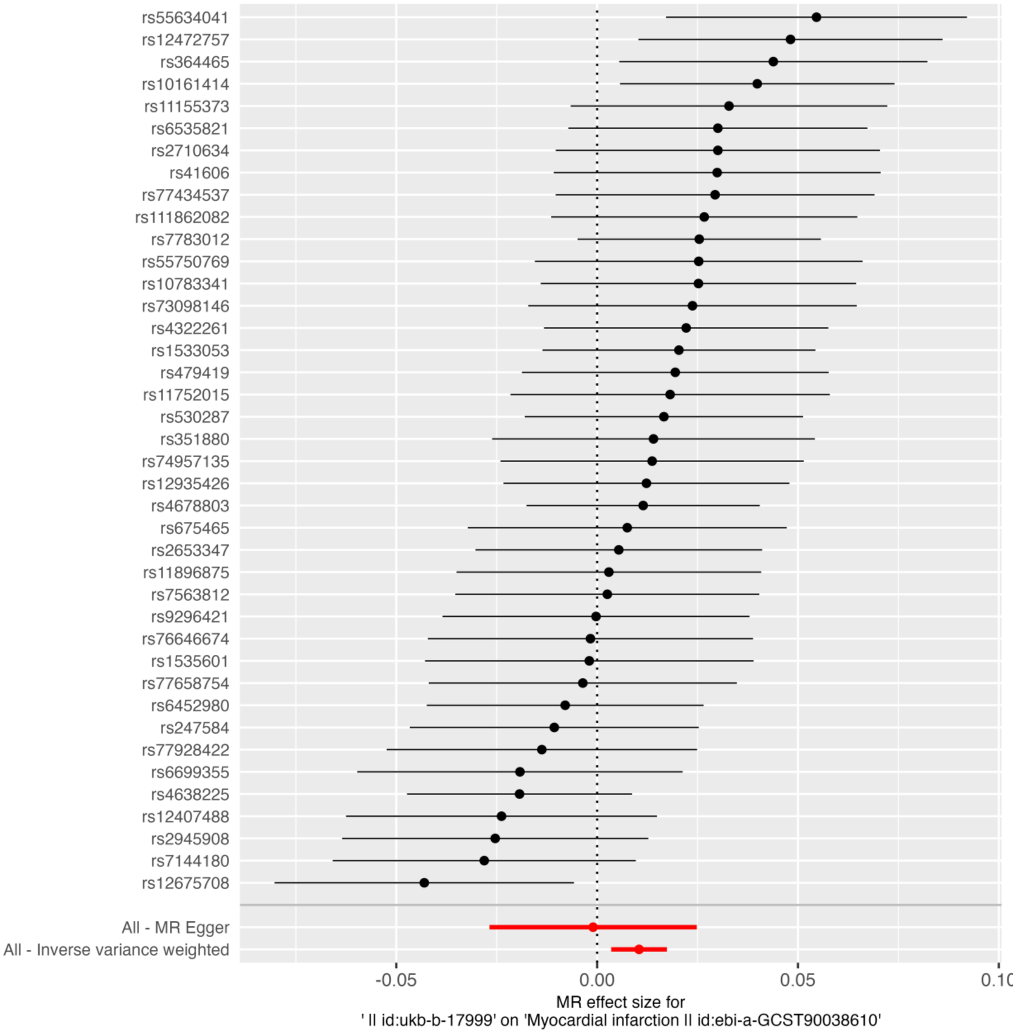

Figure S9. MR sensitivity analysis ($p < 10^{-6}$) of weekly mobile phone call duration on myocardial infarction. (a) Forest plot showing effect estimates of individual SNPs.

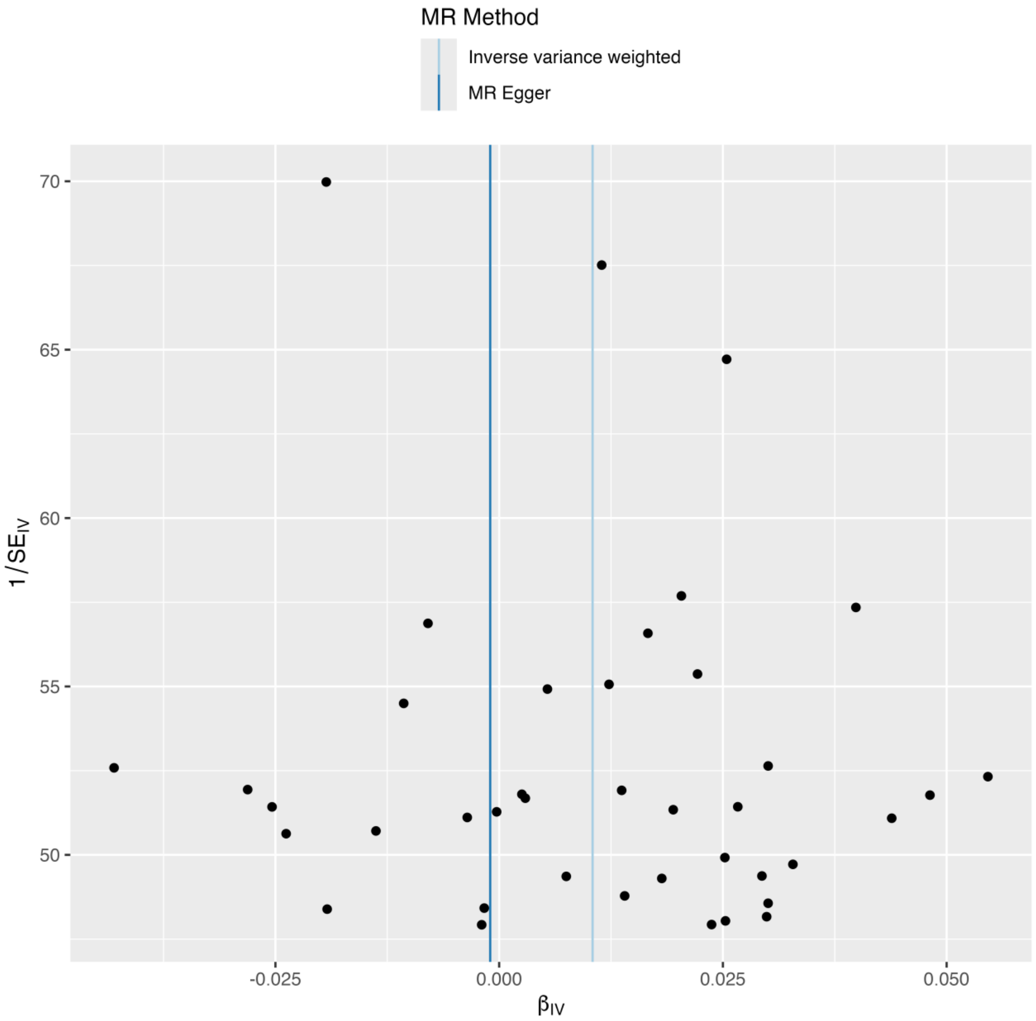

Figure S10. MR sensitivity analysis ($p < 10^{-6}$) of weekly mobile phone call duration on myocardial infarction. (b) Funnel plot assessing potential horizontal pleiotropy.

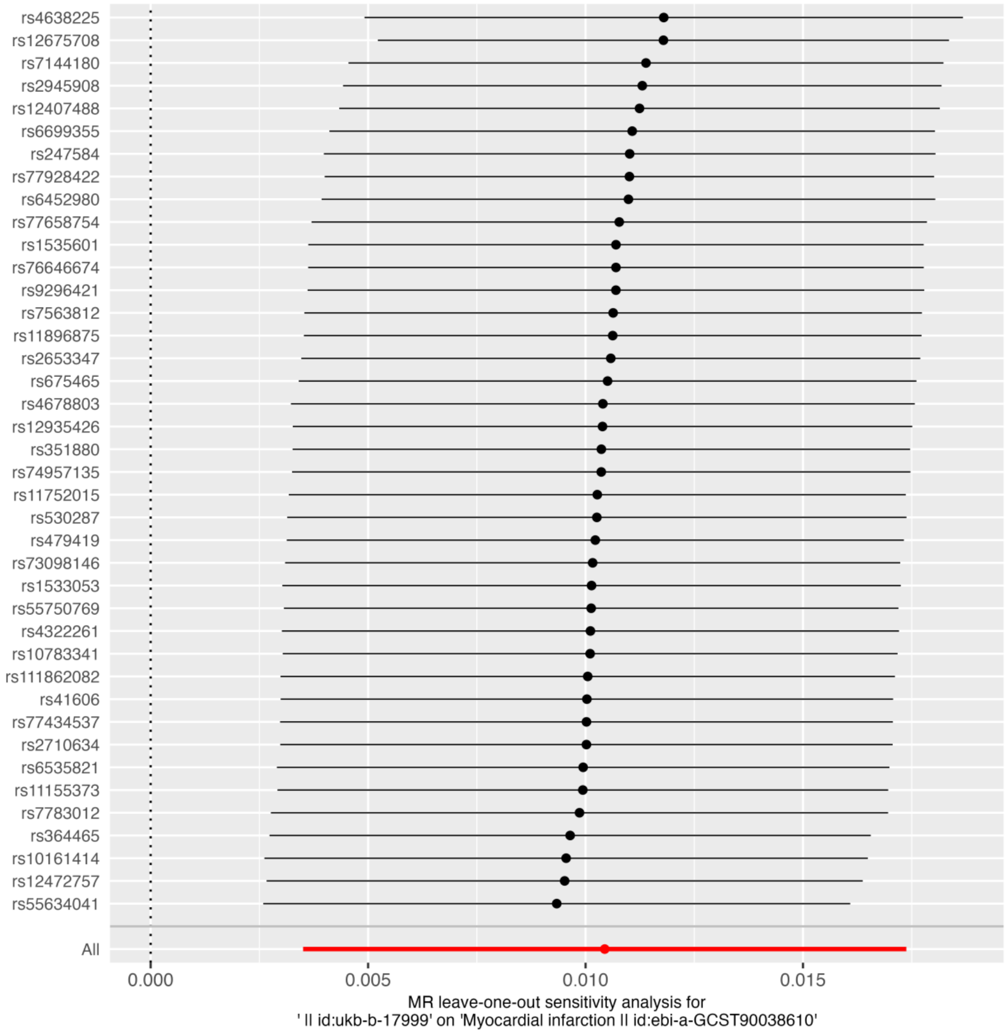

Figure S11. MR sensitivity analysis ($p < 10^{-6}$) of weekly mobile phone call duration on myocardial infarction. (c) Leave-one-out analysis to assess the influence of single SNPs on overall estimates.

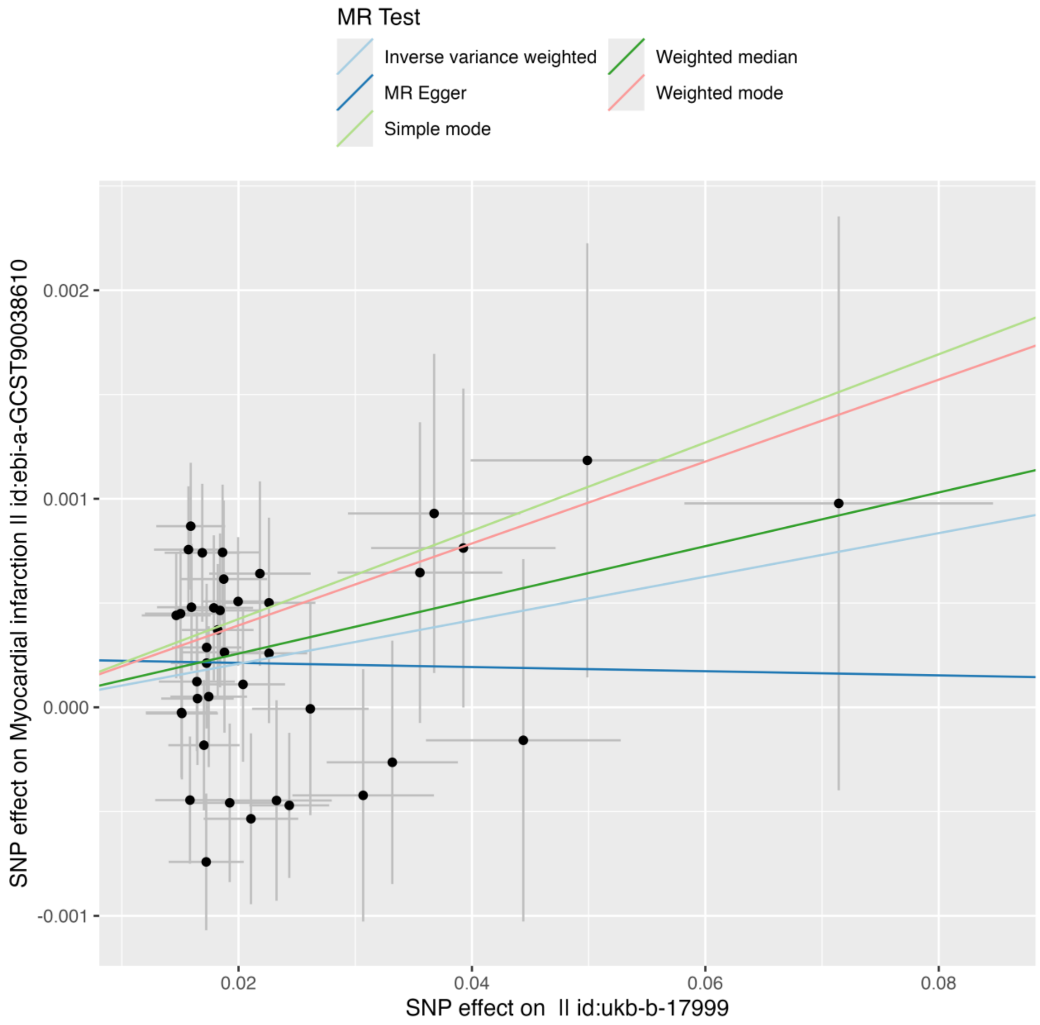

Figure S12. MR sensitivity analysis ($p < 10^{-6}$) of weekly mobile phone call duration on myocardial infarction. (d) Scatter plot of SNP effects on exposure and outcome with MR regression lines.

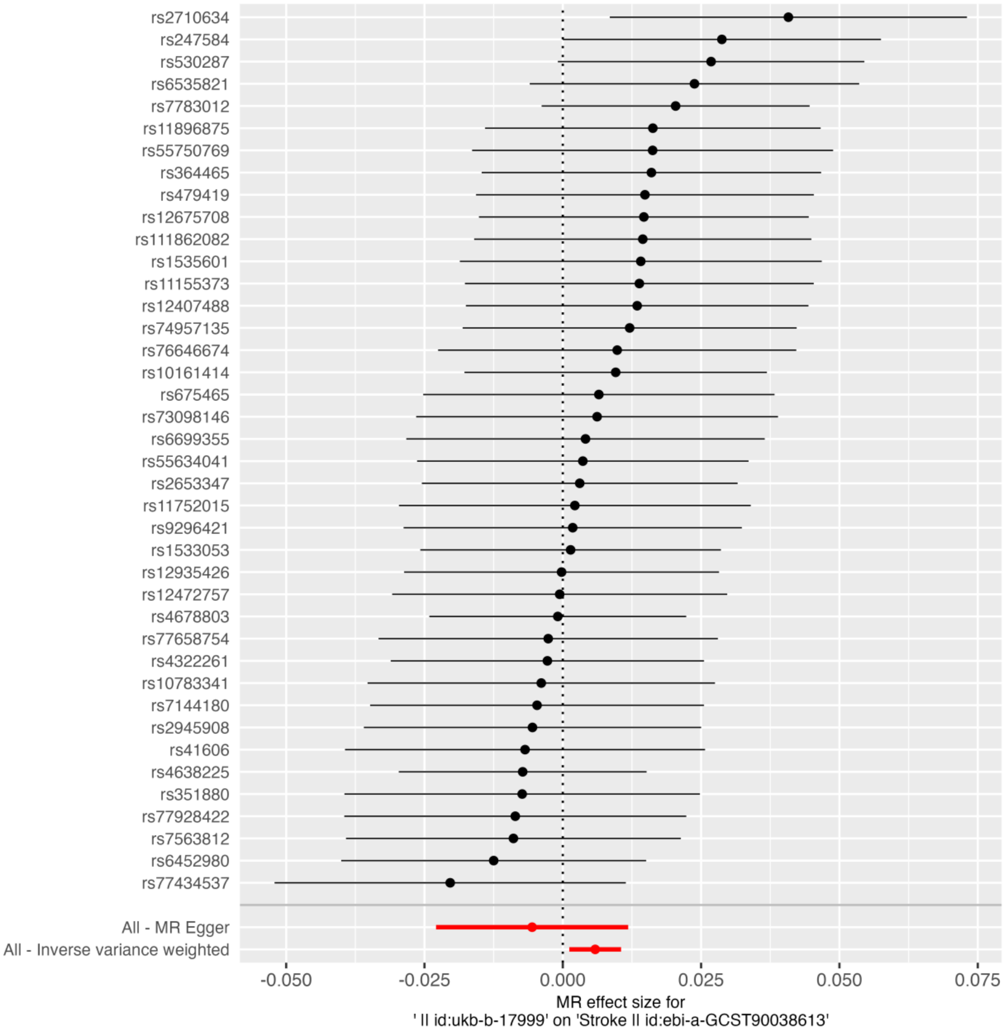

Figure S13. MR sensitivity analysis ($p < 10^{-6}$) of weekly mobile phone call duration on stroke. (a) Forest plot showing effect estimates of individual SNPs.

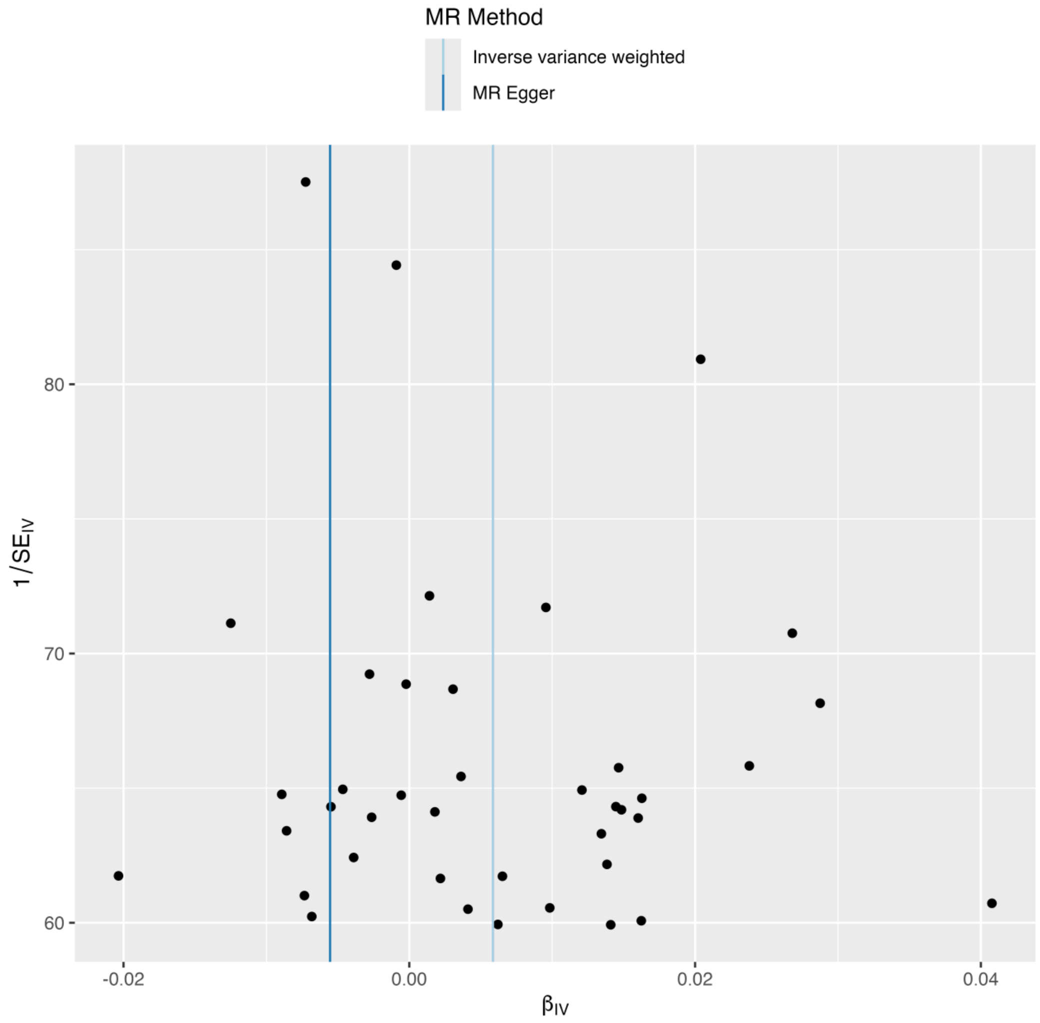

Figure S14. MR sensitivity analysis ($p < 10^{-6}$) of weekly mobile phone call duration on stroke. (b) Funnel plot assessing potential horizontal pleiotropy.

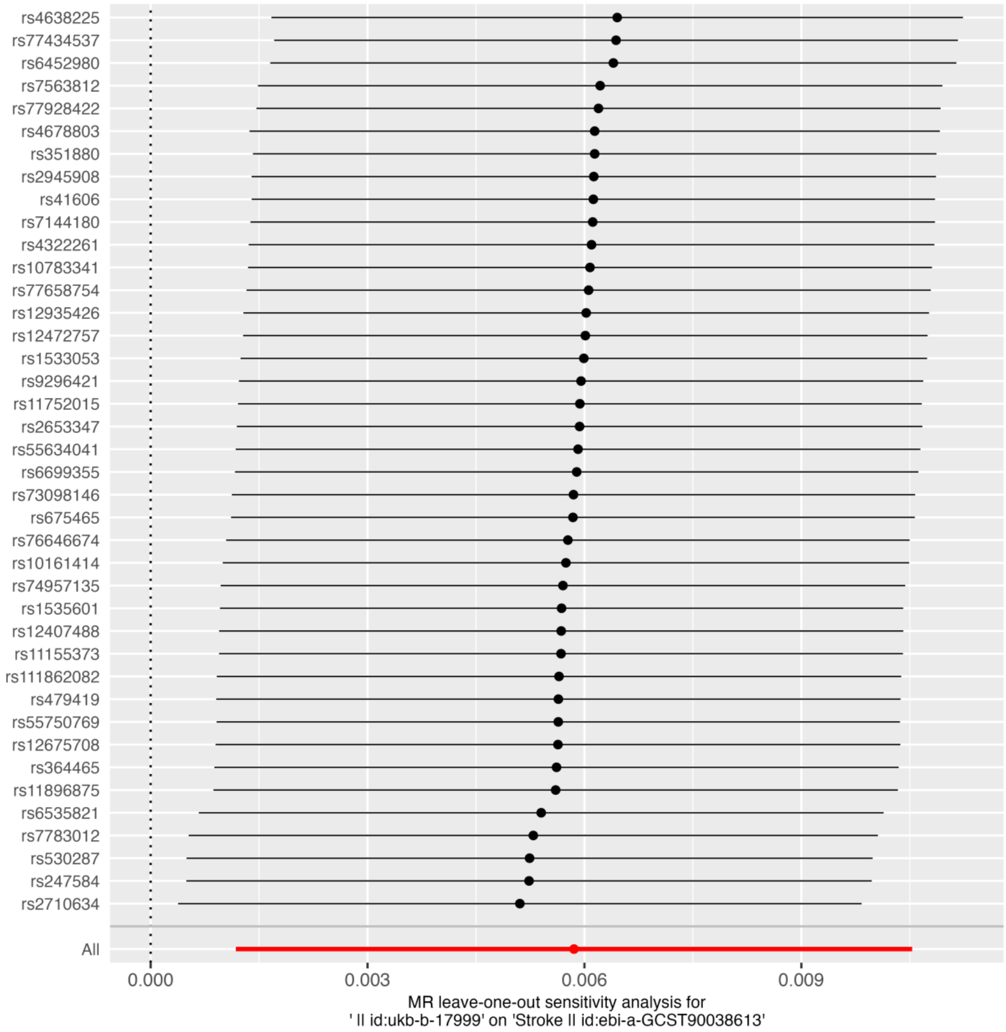

Figure S15. MR sensitivity analysis ($p < 10^{-6}$) of weekly mobile phone call duration on stroke. (c) Leave-one-out analysis to assess the influence of single SNPs on overall estimates.

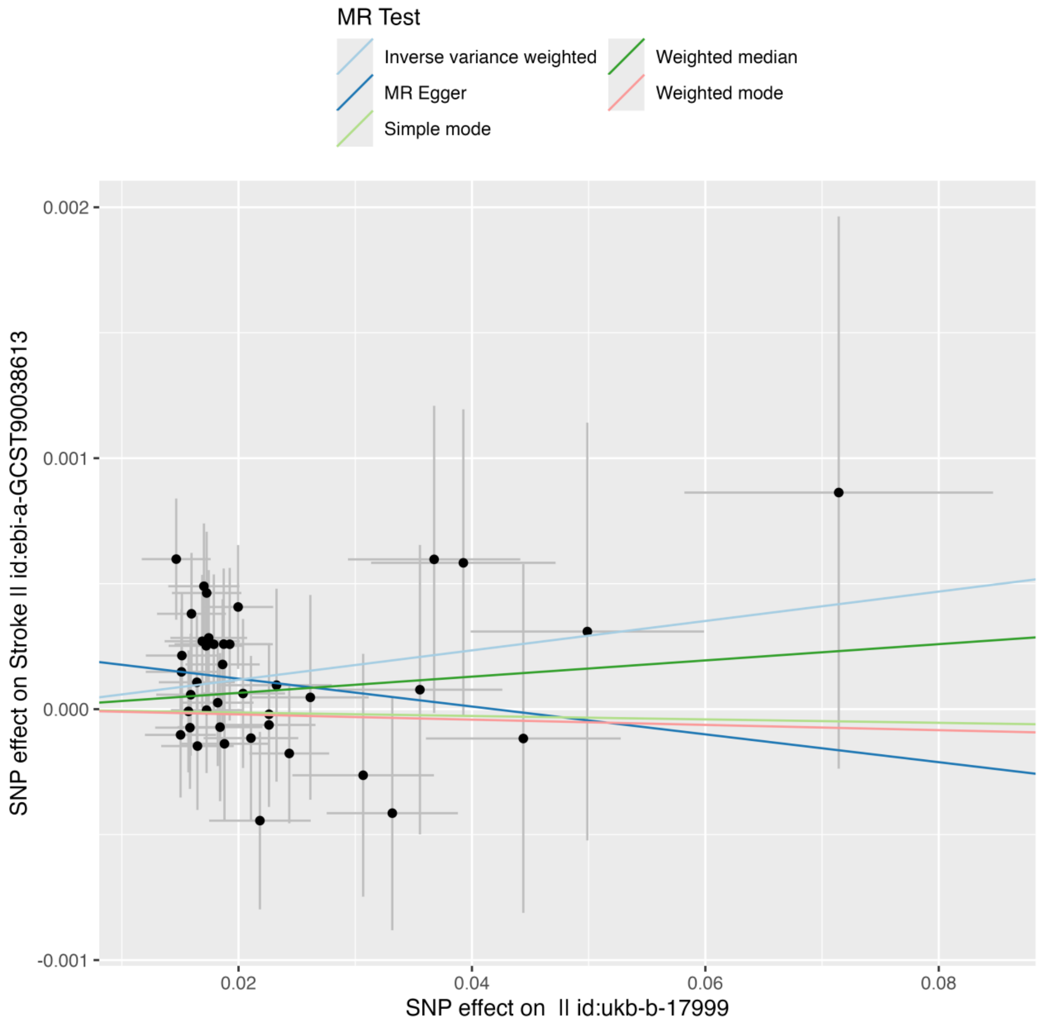

Figure S16. MR sensitivity analysis ($p < 10^{-6}$) of weekly mobile phone call duration on stroke. (d) Scatter plot of SNP effects on exposure and outcome with MR regression lines.

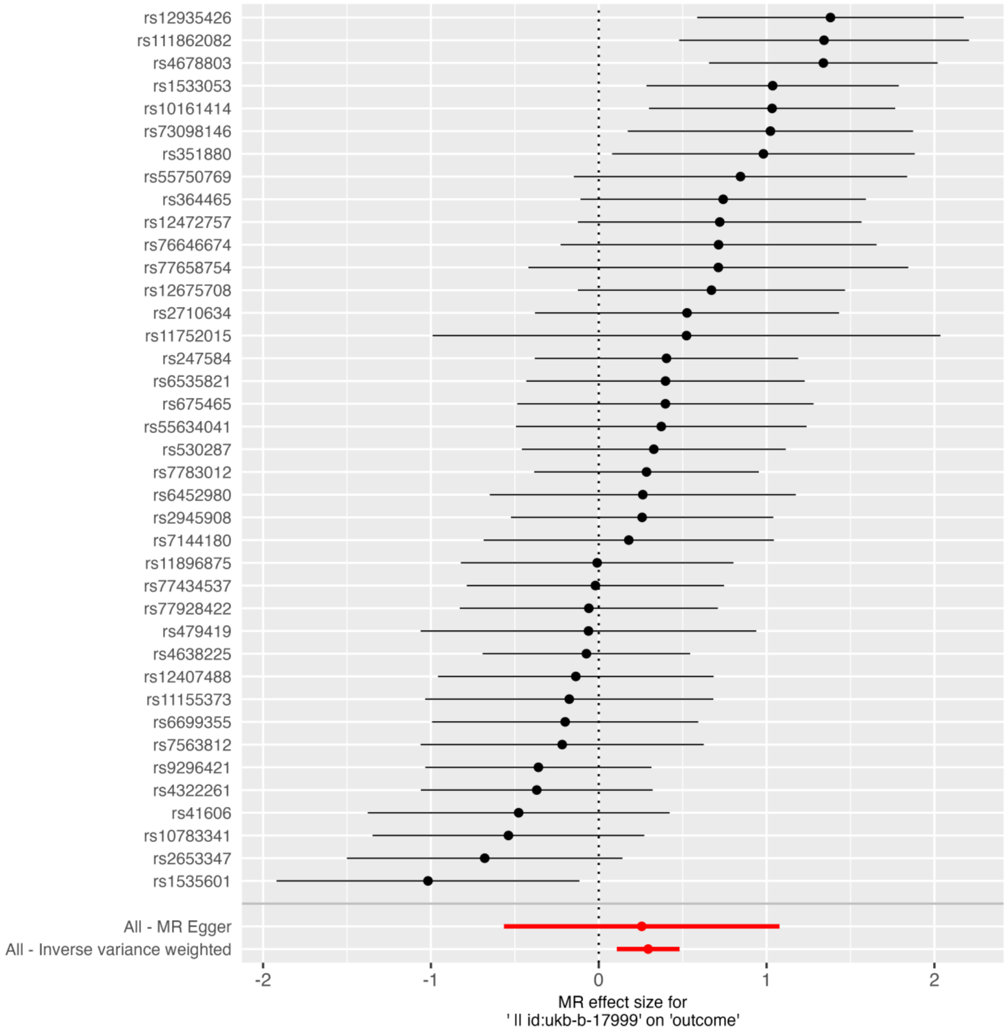

Figure S17. MR sensitivity analysis ($p < 10^{-6}$) of weekly mobile phone call duration on major depressive disorder. (a) Forest plot showing effect estimates of individual SNPs.

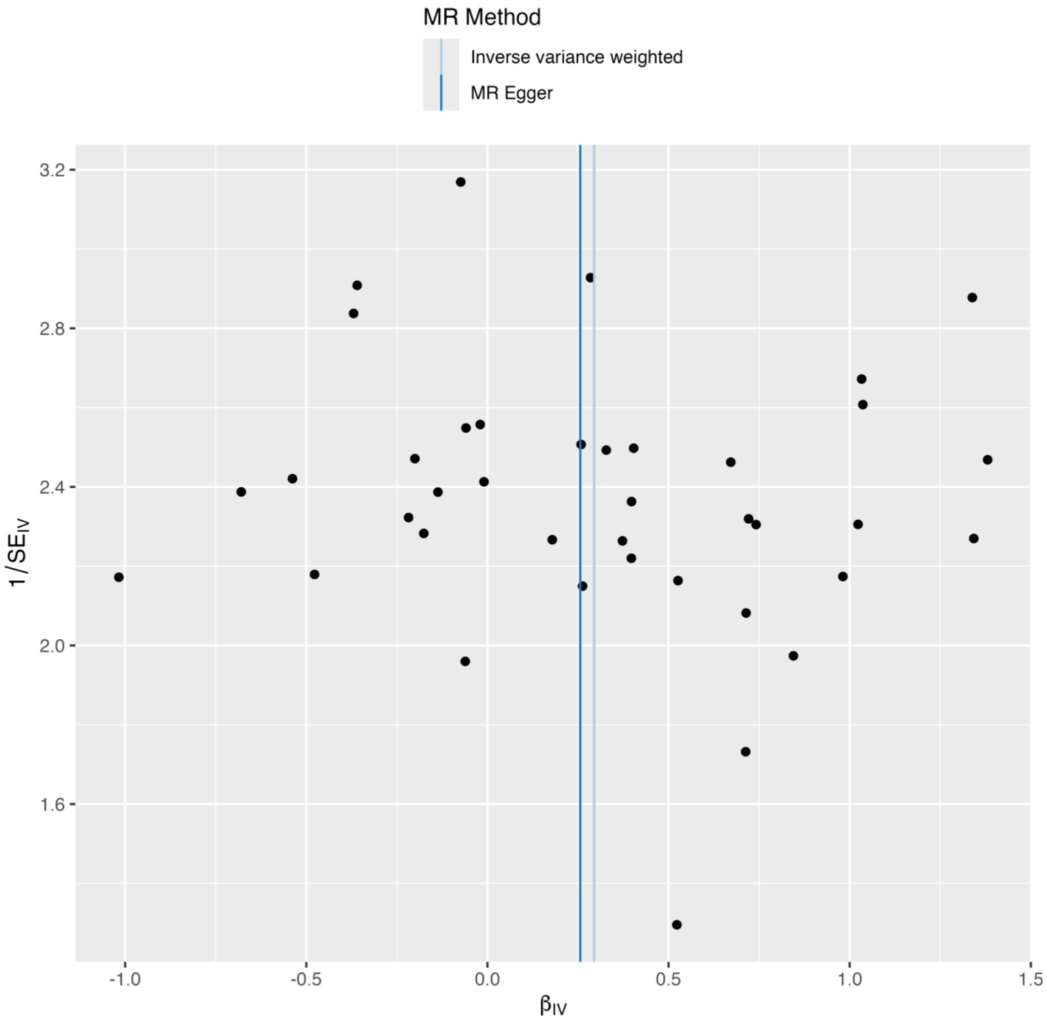

Figure S18. MR sensitivity analysis ($p < 10^{-6}$) of weekly mobile phone call duration on major depressive disorder. (b) Funnel plot assessing potential horizontal pleiotropy.

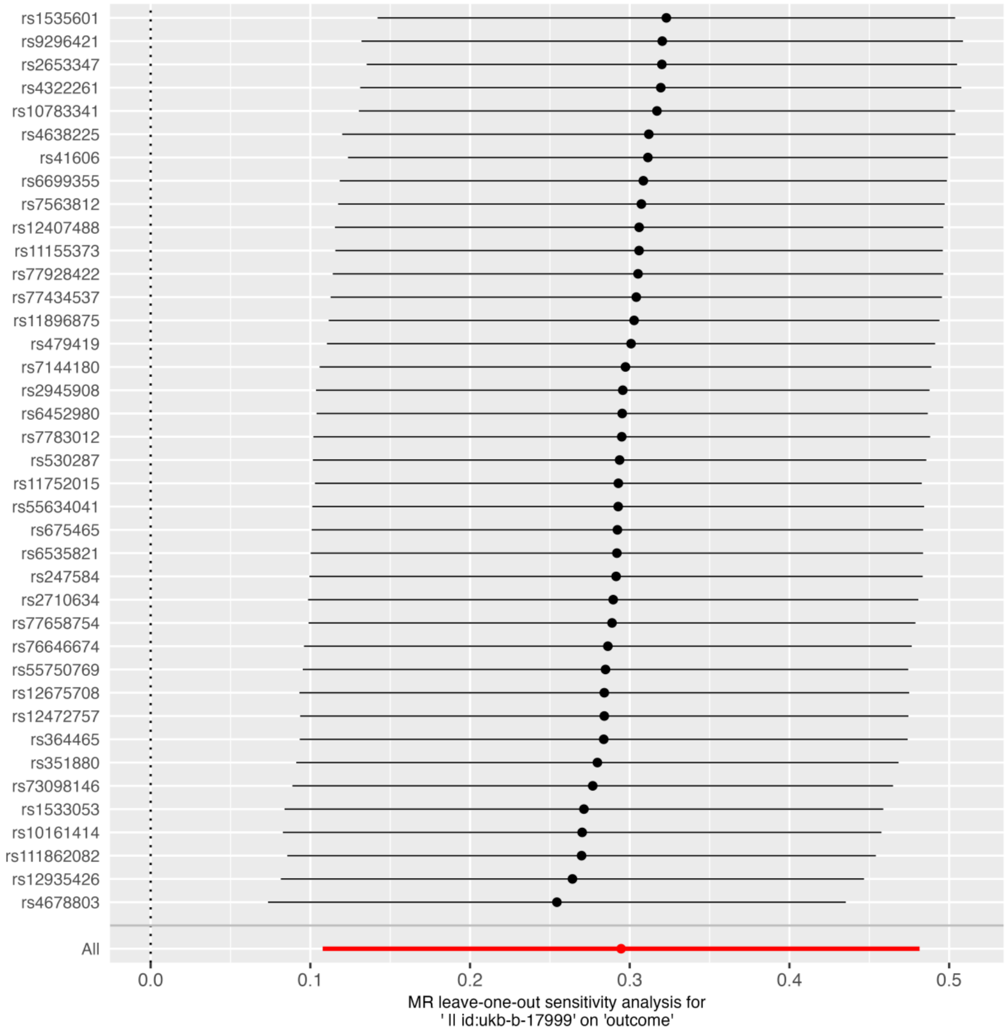

Figure S19. MR sensitivity analysis ($p < 10^{-6}$) of weekly mobile phone call duration on major depressive disorder. (c) Leave-one-out analysis to assess the influence of single SNPs on overall estimates.

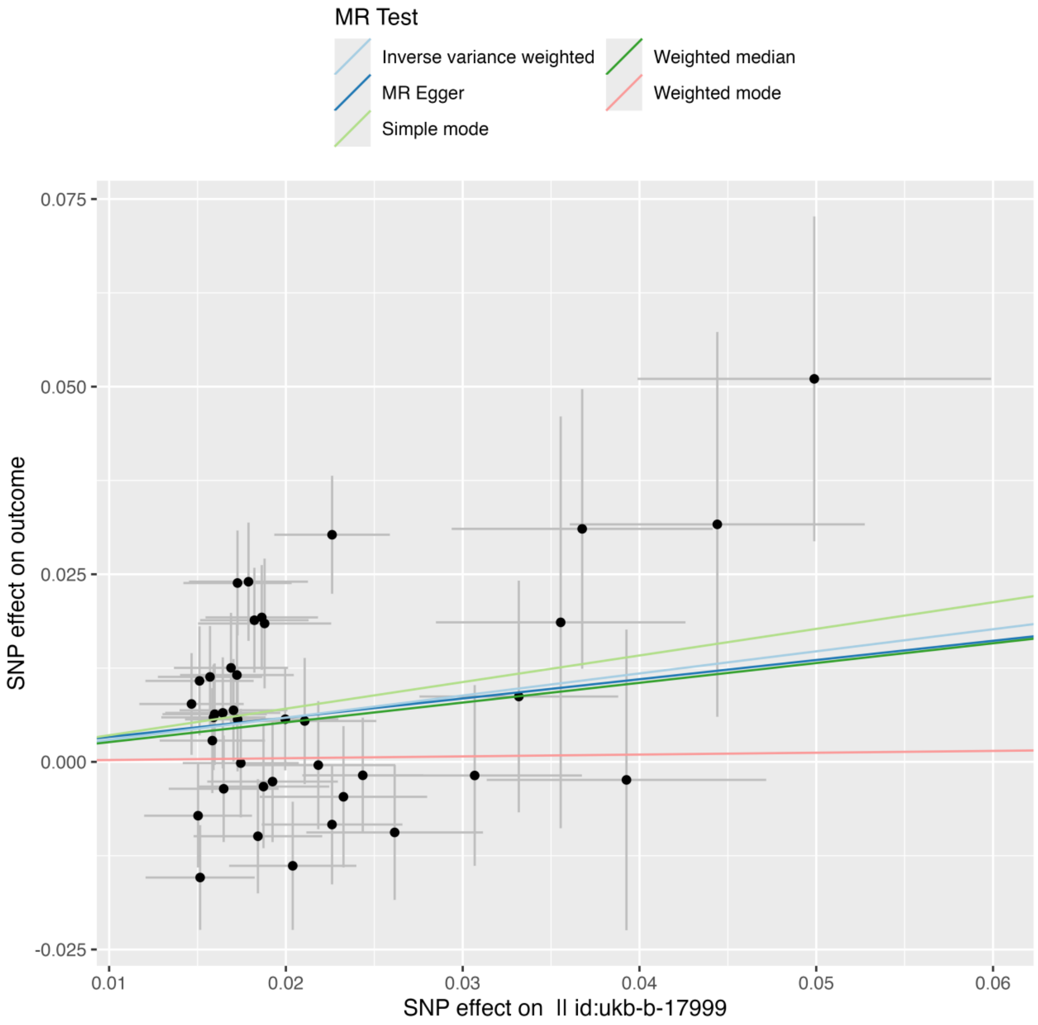

Figure S20. MR sensitivity analysis ($p < 10^{-6}$) of weekly mobile phone call duration on major depressive disorder. (d) Scatter plot of SNP effects on exposure and outcome with MR regression lines.

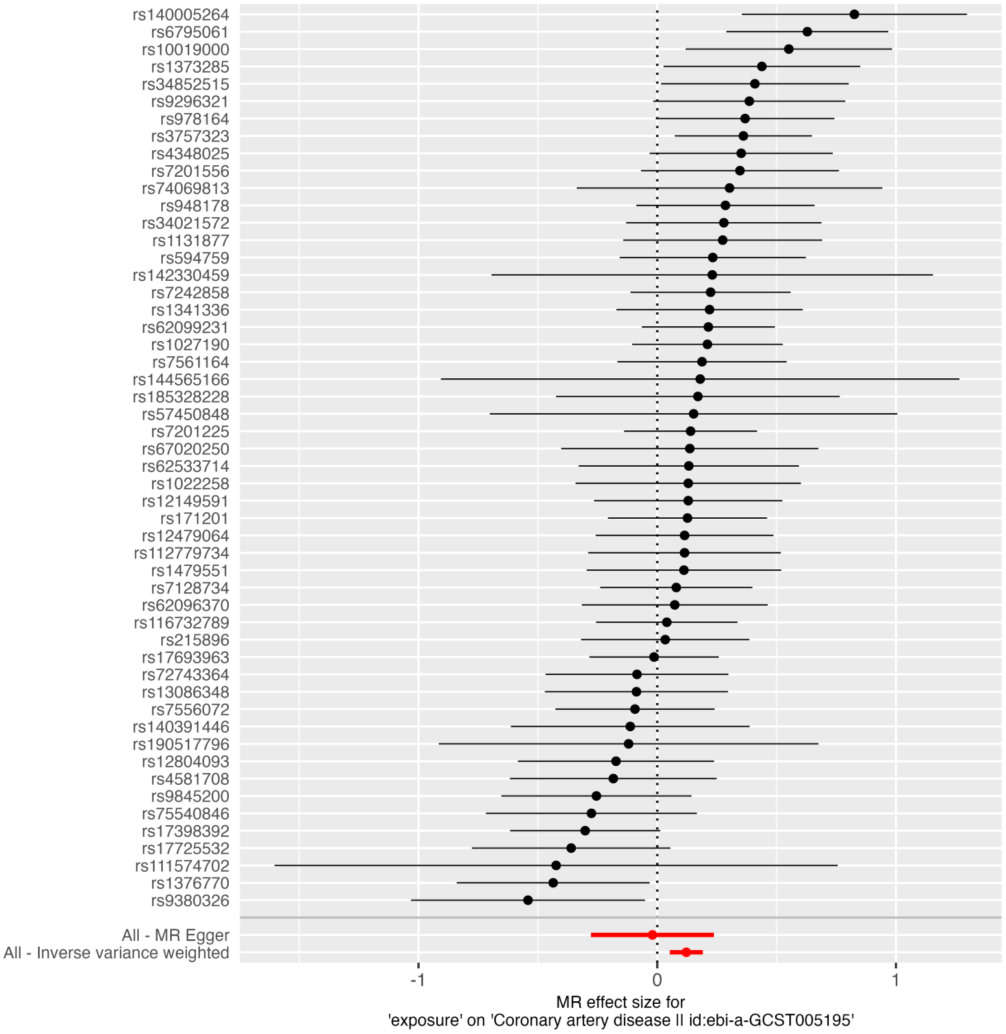

Figure S21. MR sensitivity analysis ($p < 10^{-6}$) of major depressive disorder on coronary artery disease. (a) Forest plot showing effect estimates of individual SNPs.

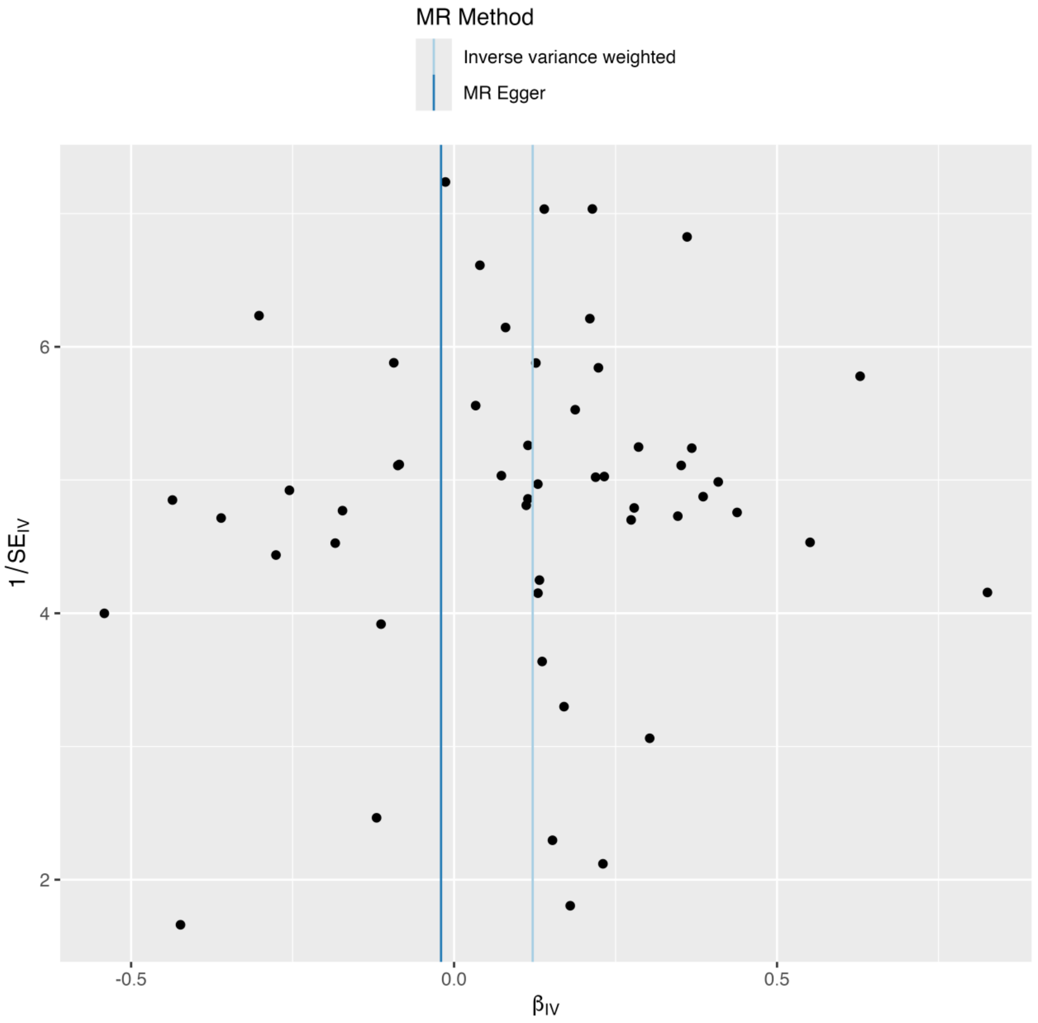

Figure S22. MR sensitivity analysis ($p < 10^{-6}$) of major depressive disorder on coronary artery disease. (b) Funnel plot assessing potential horizontal pleiotropy.

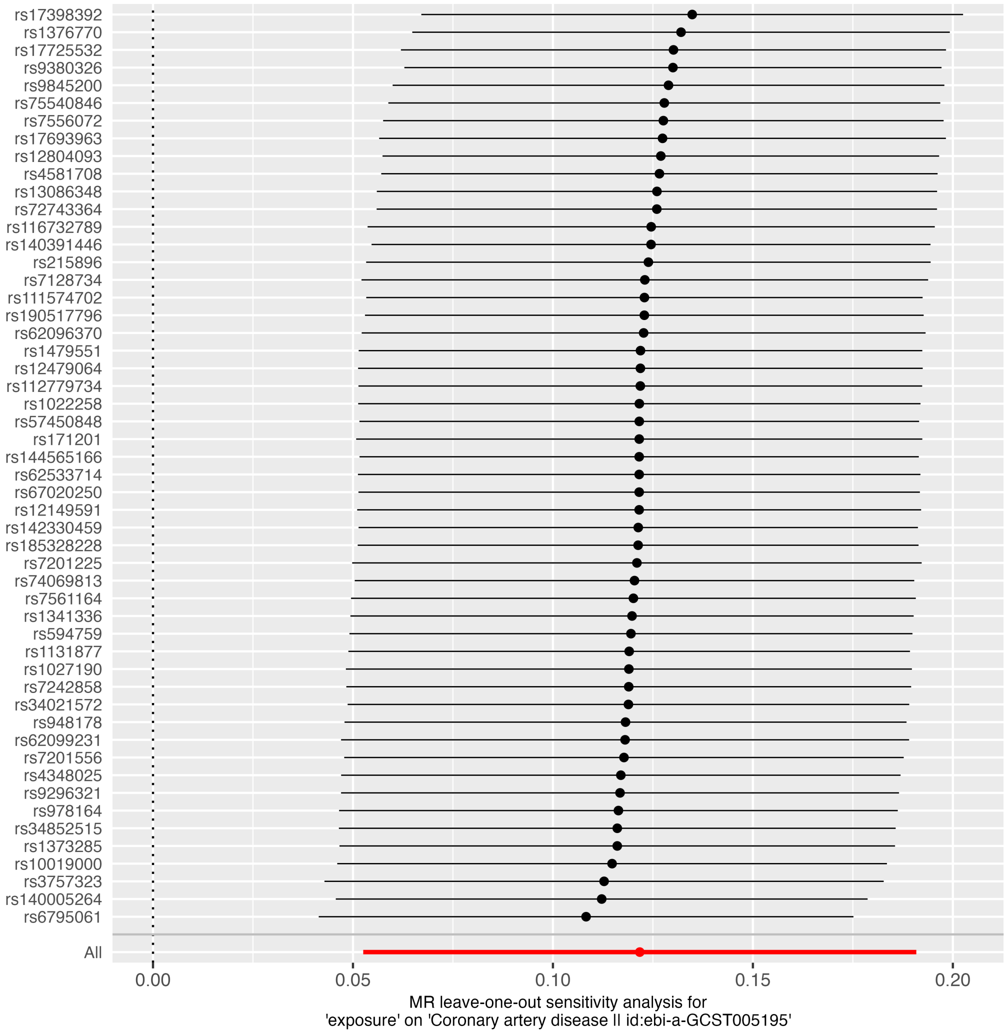

Figure S23. MR sensitivity analysis ($p < 10^{-6}$) of major depressive disorder on coronary artery disease. (c) Leave-one-out analysis to assess the influence of single SNPs on overall estimates.

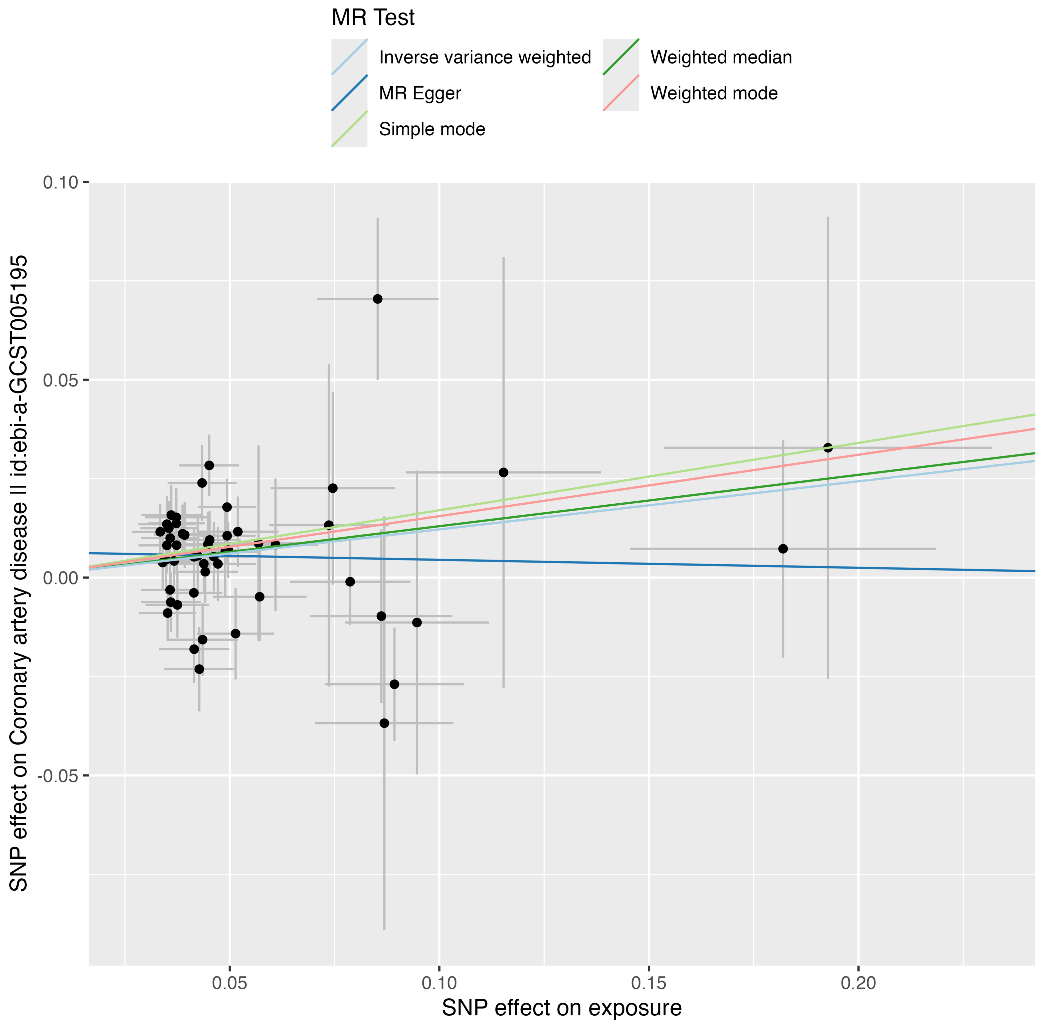

Figure S24. MR sensitivity analysis ($p < 10^{-6}$) of major depressive disorder on coronary artery disease. (d) Scatter plot of SNP effects on exposure and outcome with MR regression lines.

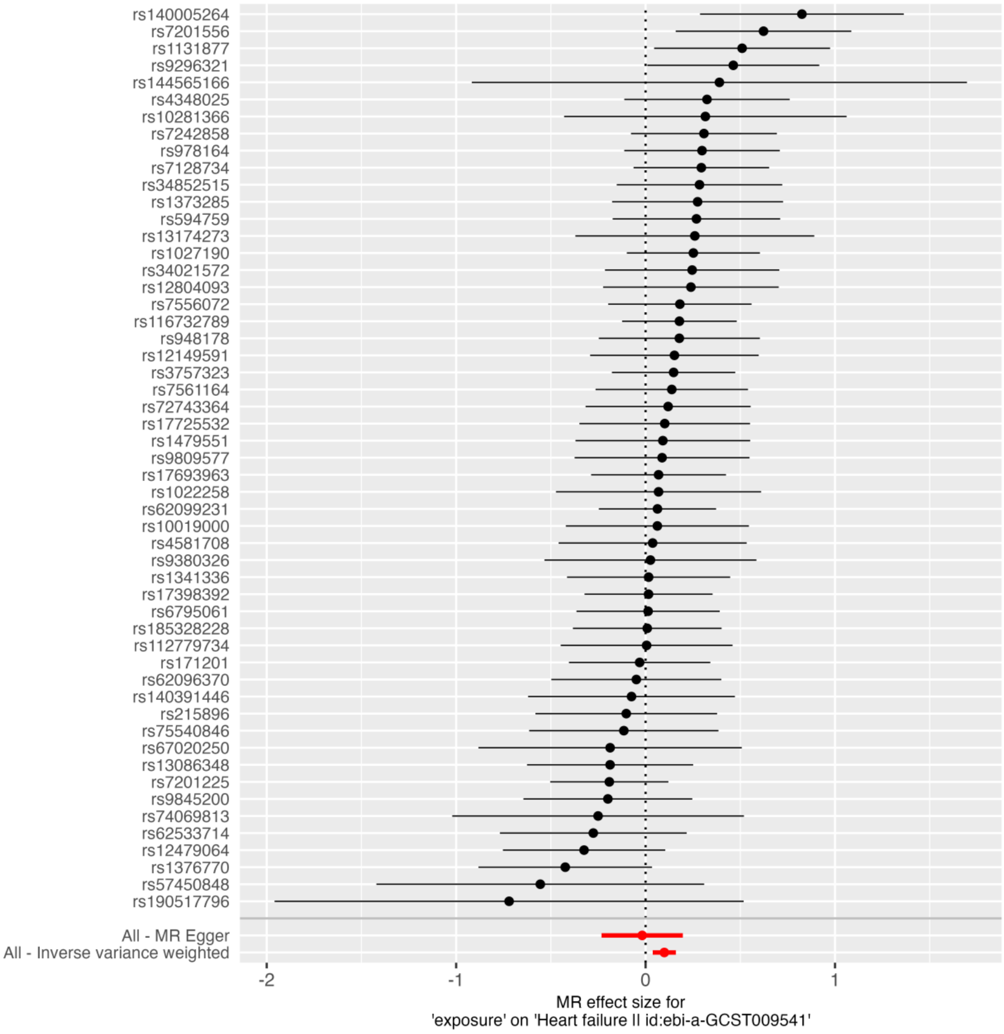

Figure S25. MR sensitivity analysis ($p < 10^{-6}$) of major depressive disorder on heart failure. (a) Forest plot showing effect estimates of individual SNPs.

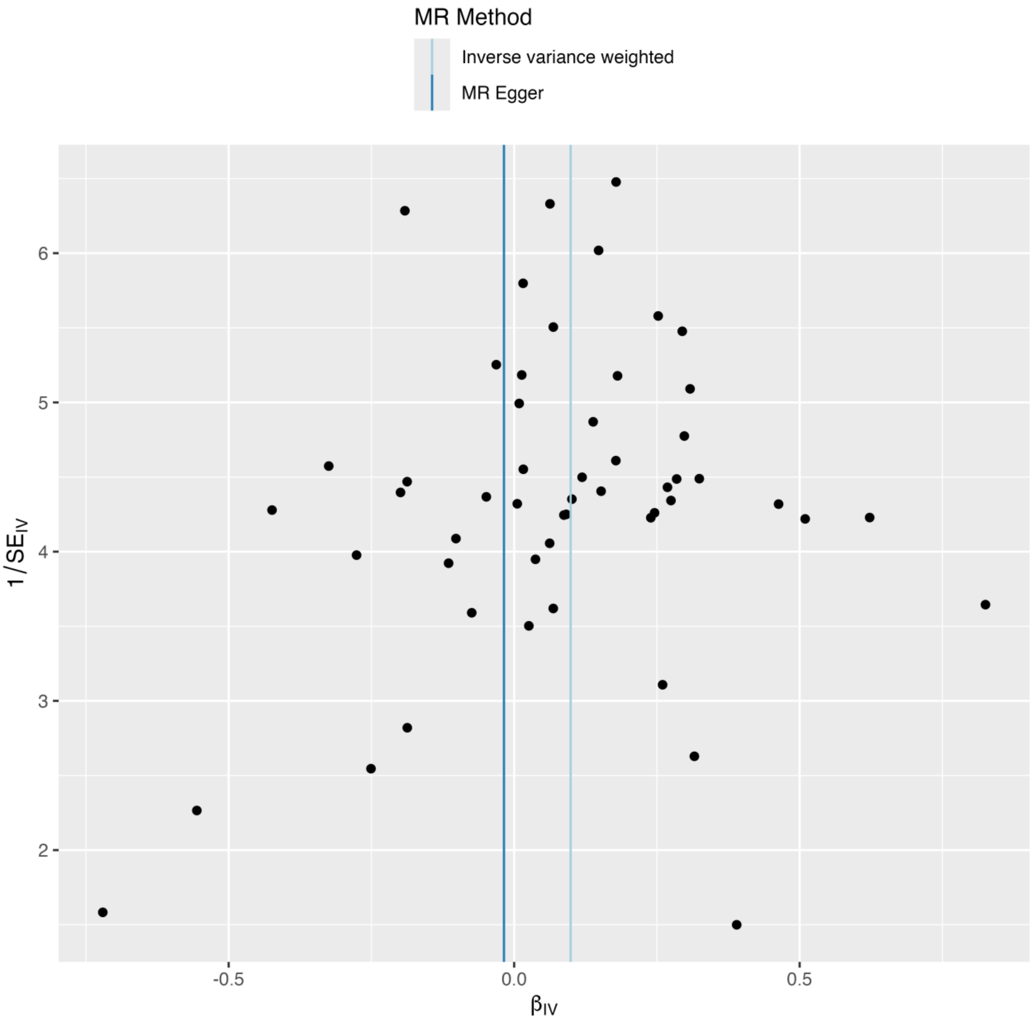

Figure S26. MR sensitivity analysis ($p < 10^{-6}$) of major depressive disorder on heart failure. (b) Funnel plot assessing potential horizontal pleiotropy.

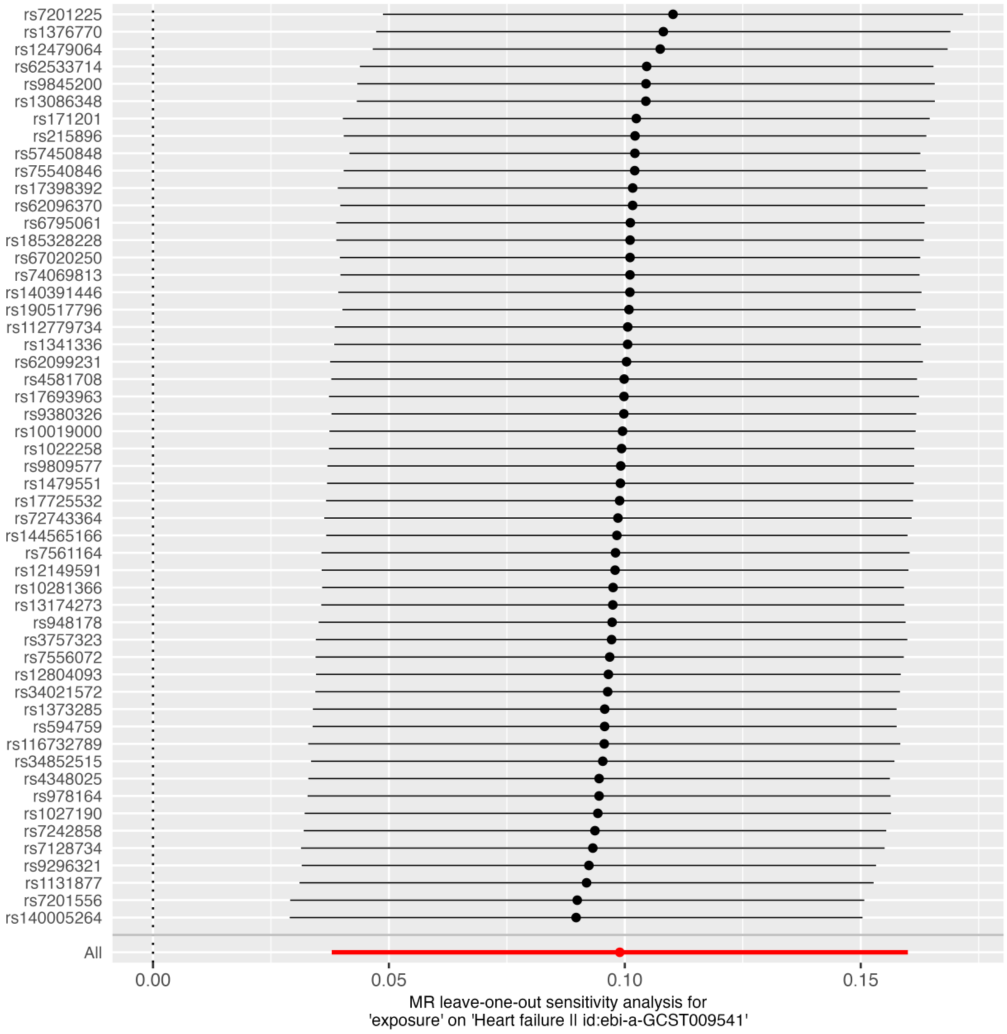

Figure S27. MR sensitivity analysis ($p < 10^{-6}$) of major depressive disorder on heart failure. (c) Leave-one-out analysis to assess the influence of single SNPs on overall estimates.

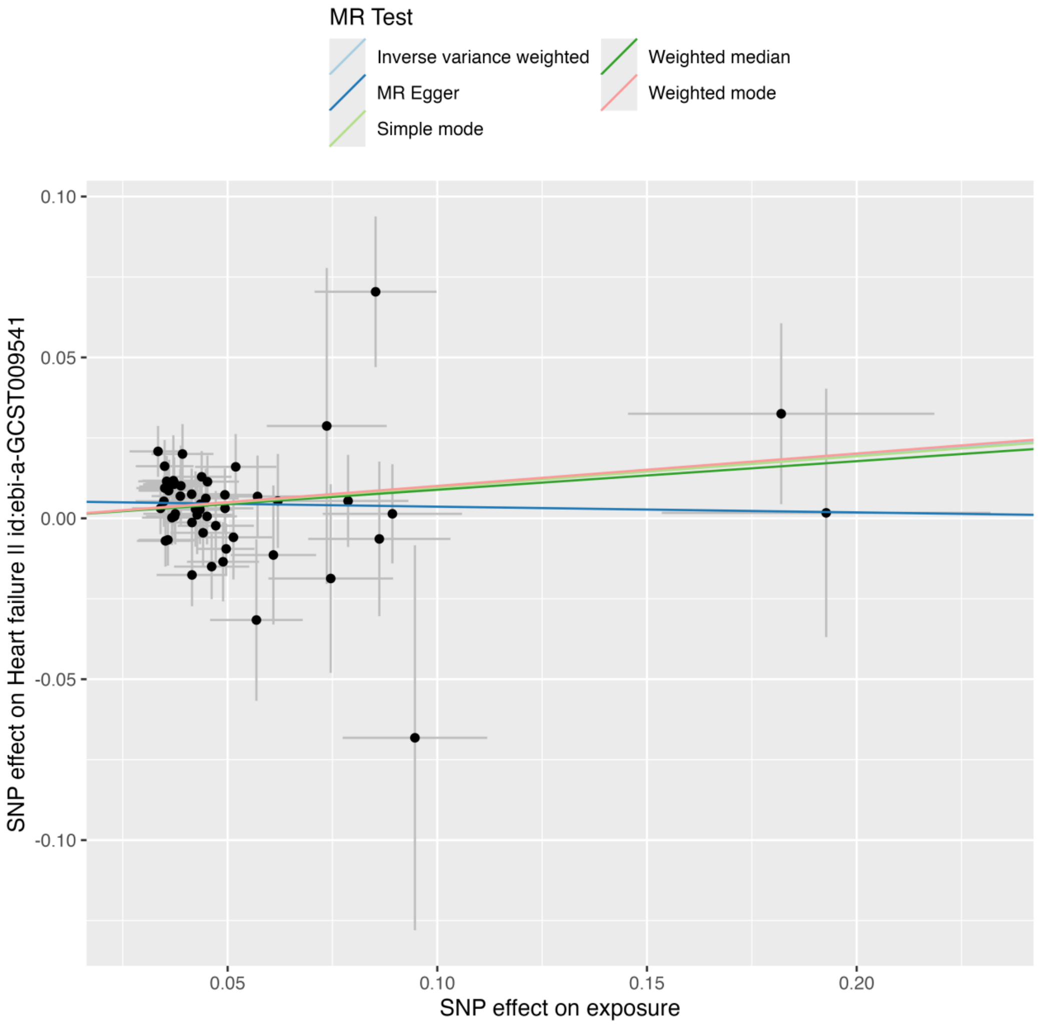

Figure S28. MR sensitivity analysis ($p < 10^{-6}$) of major depressive disorder on heart failure. (d) Scatter plot of SNP effects on exposure and outcome with MR regression lines.

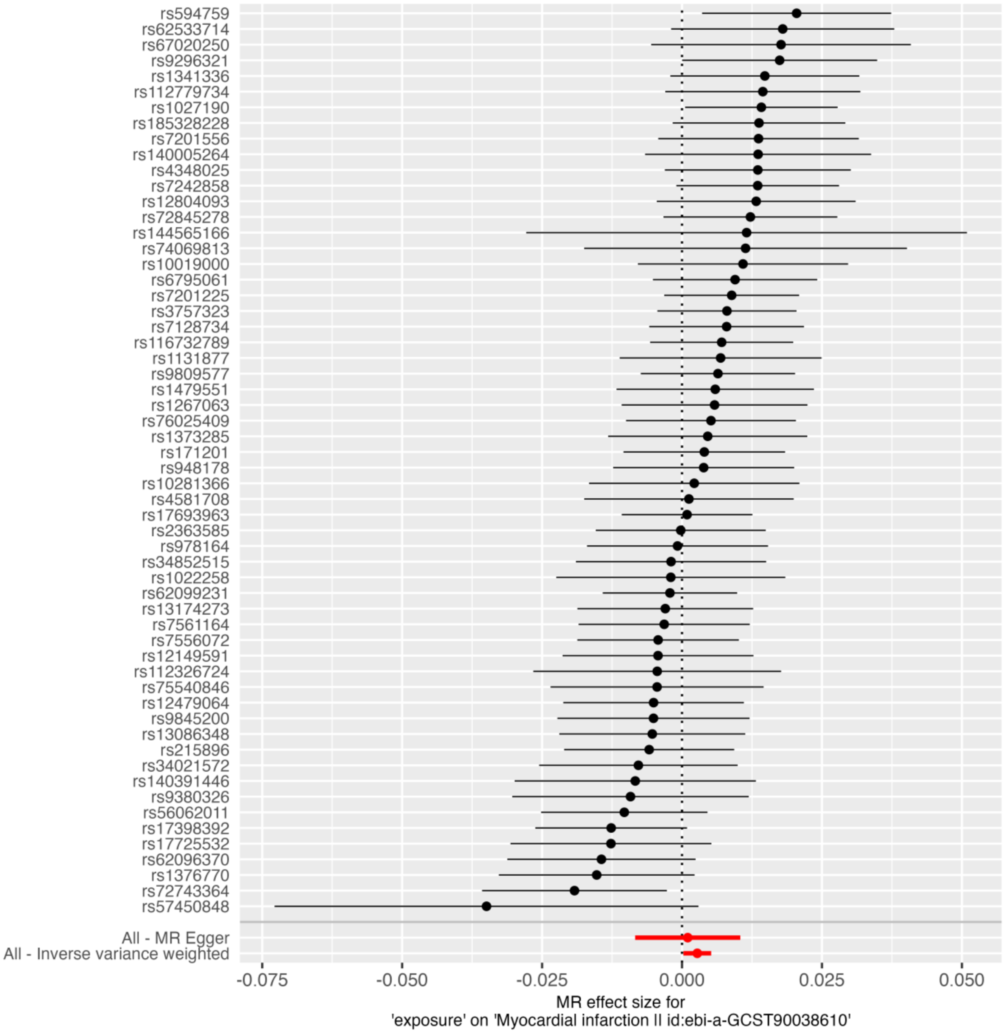

Figure S29. MR sensitivity analysis ($p < 10^{-6}$) of major depressive disorder on myocardial infarction. (a) Forest plot showing effect estimates of individual SNPs.

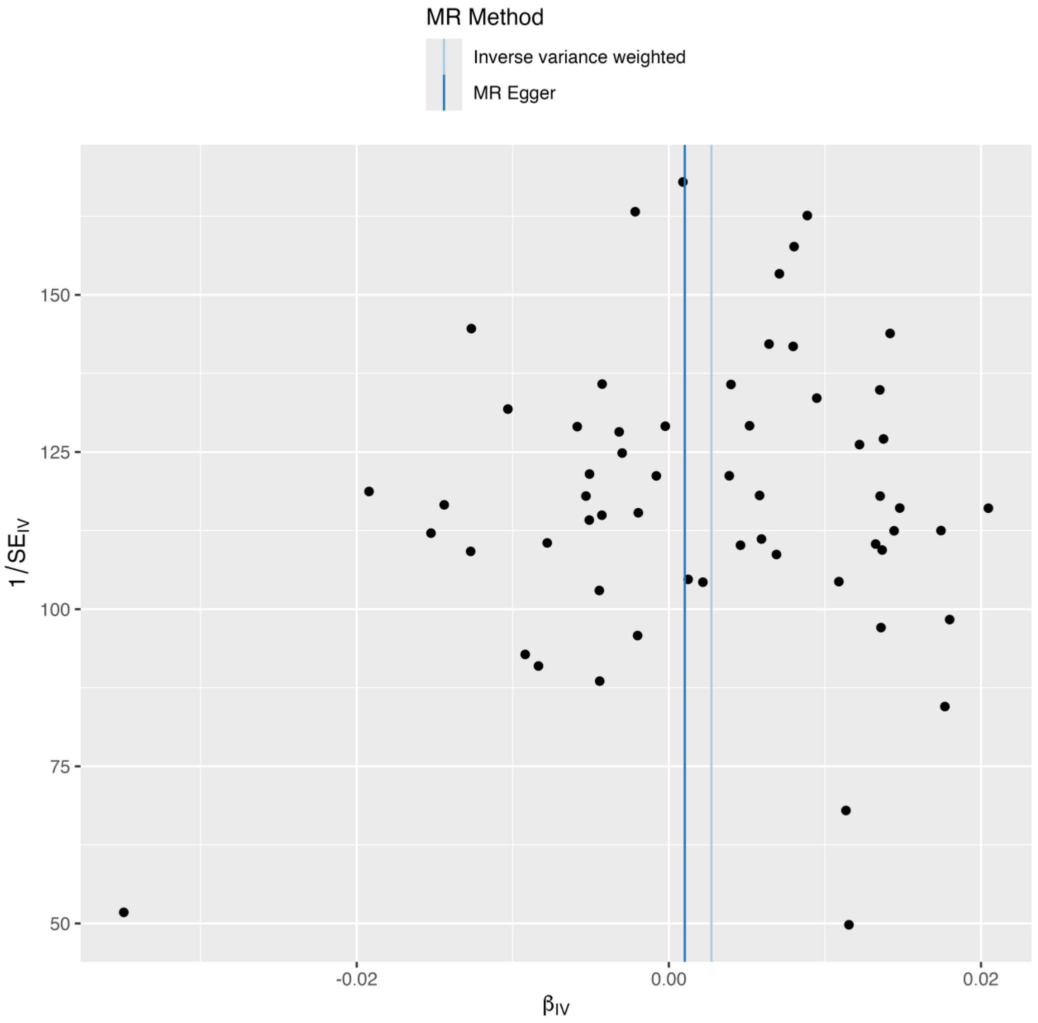

Figure S30. MR sensitivity analysis ($p < 10^{-6}$) of major depressive disorder on myocardial infarction. (b) Funnel plot assessing potential horizontal pleiotropy.

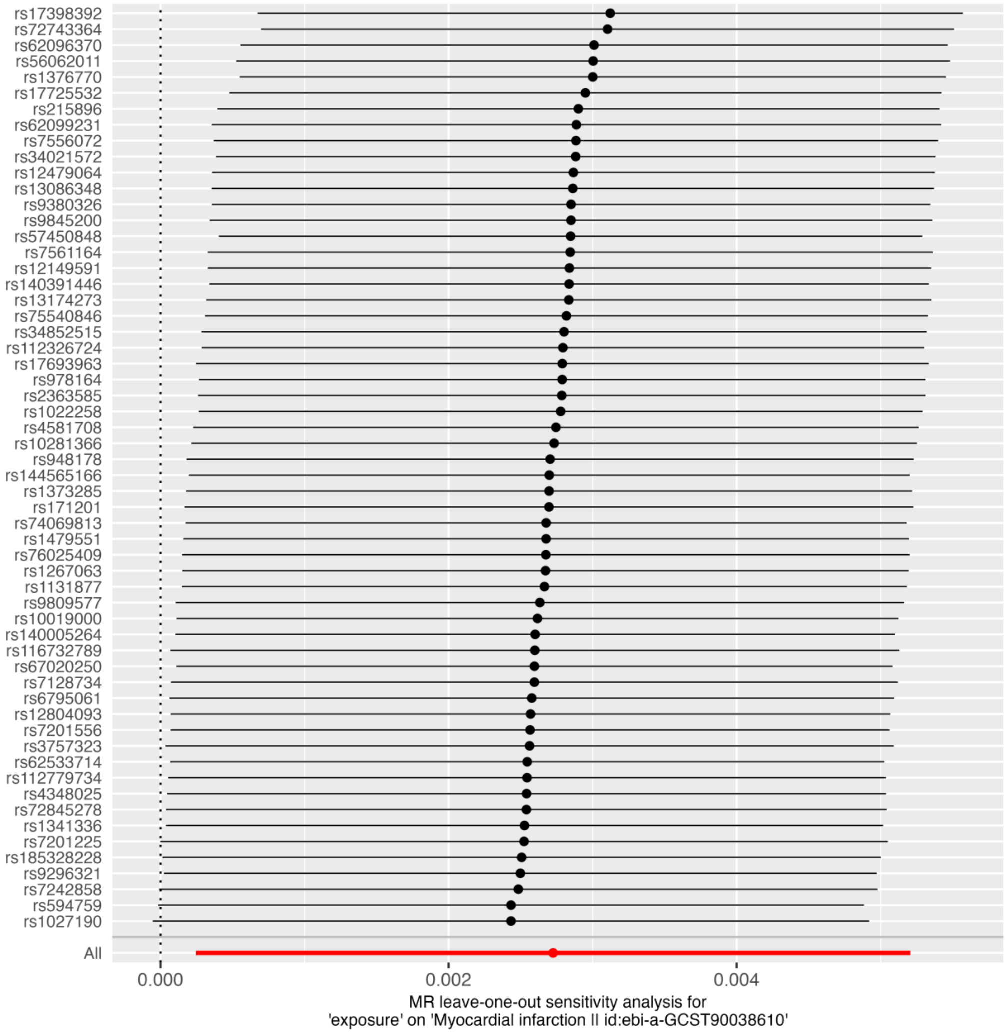

Figure S31. MR sensitivity analysis ($p < 10^{-6}$) of major depressive disorder on myocardial infarction. (c) Leave-one-out analysis to assess the influence of single SNPs on overall estimates.

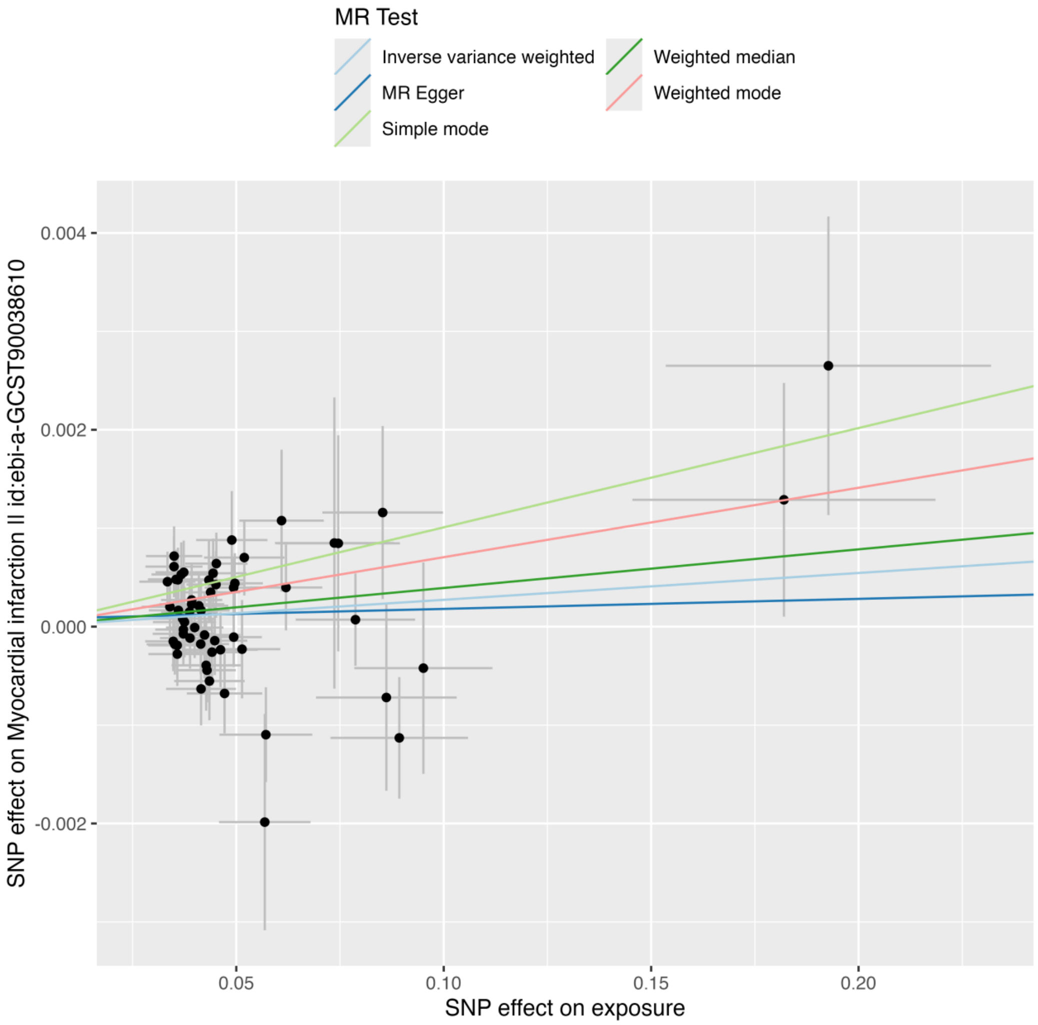

Figure S32. MR sensitivity analysis ($p < 10^{-6}$) of major depressive disorder on myocardial infarction. (d) Scatter plot of SNP effects on exposure and outcome with MR regression lines.

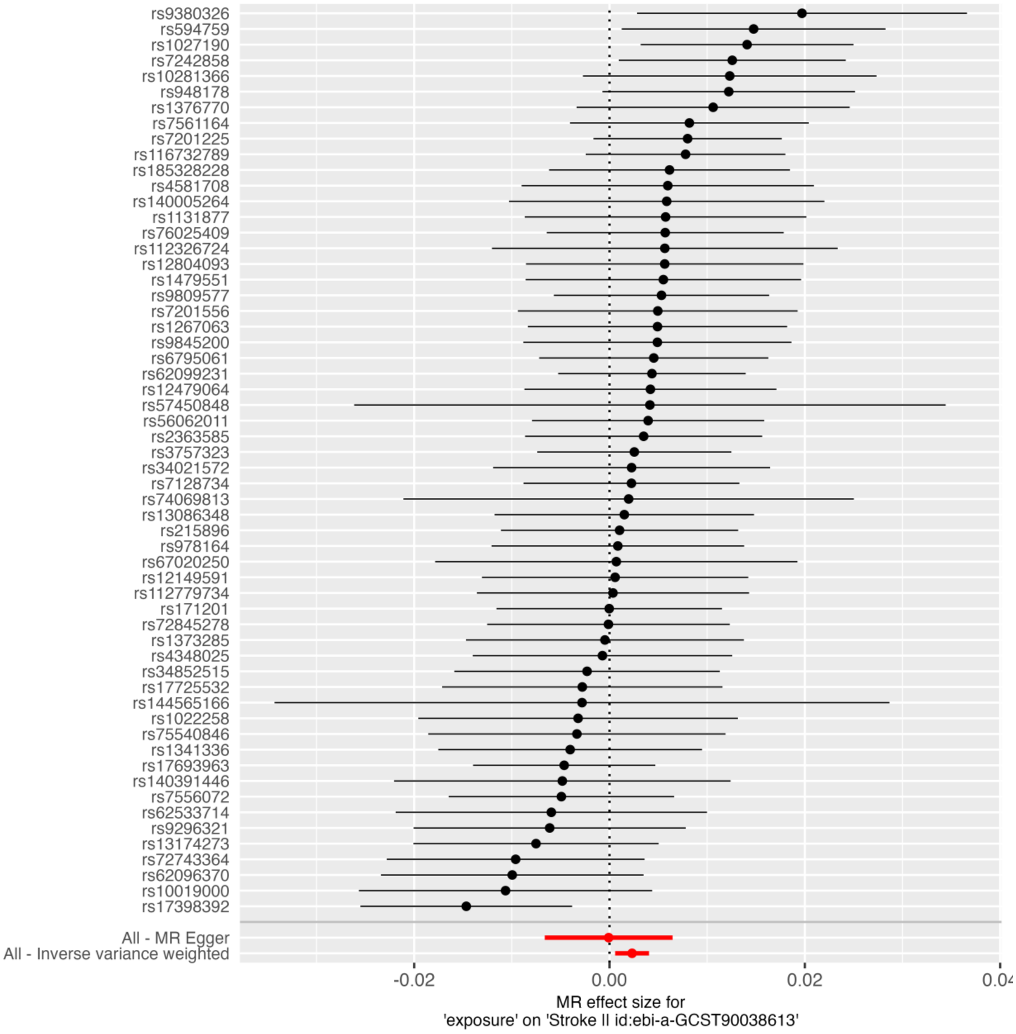

Figure S33. MR sensitivity analysis ($p < 10^{-6}$) of major depressive disorder on stroke. (a) Forest plot showing effect estimates of individual SNPs.

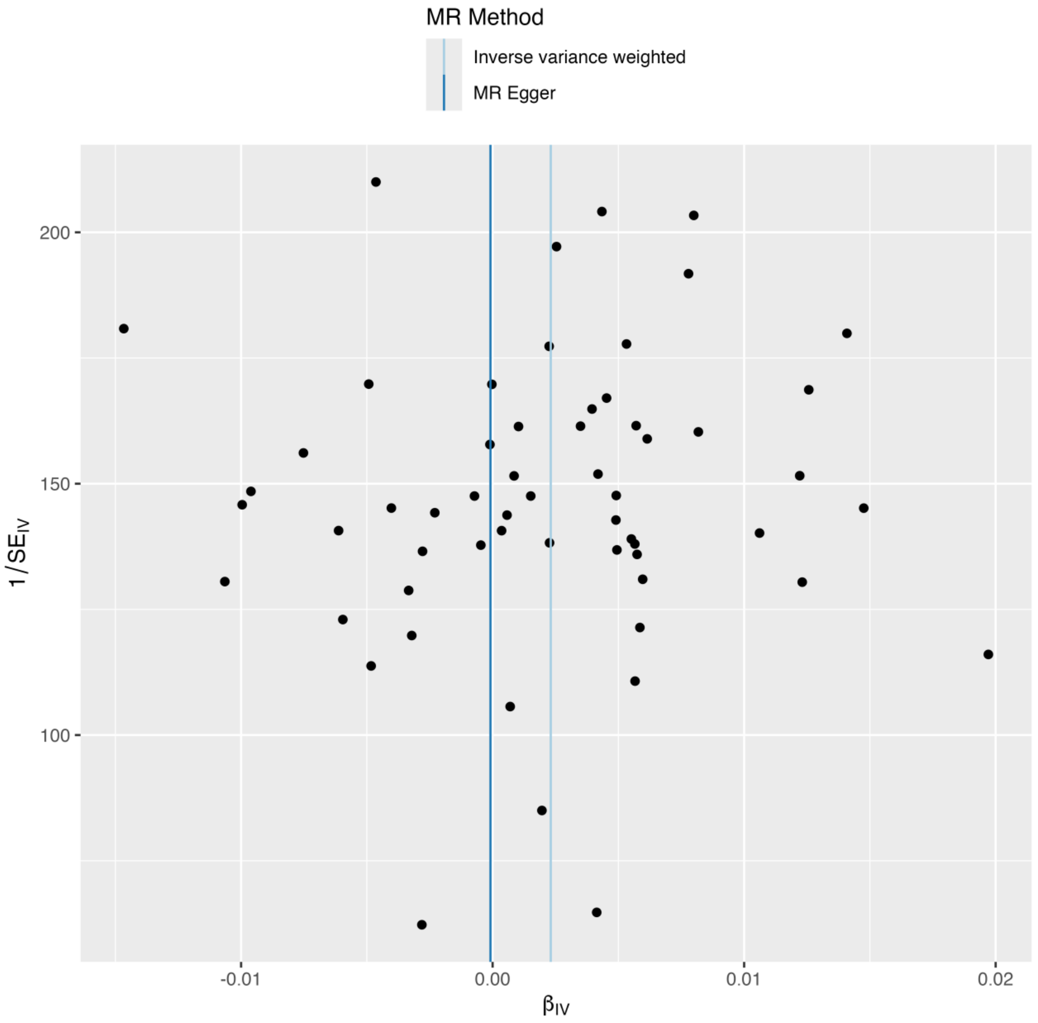

Figure S34. MR sensitivity analysis ($p < 10^{-6}$) of major depressive disorder on stroke. (b) Funnel plot assessing potential horizontal pleiotropy.

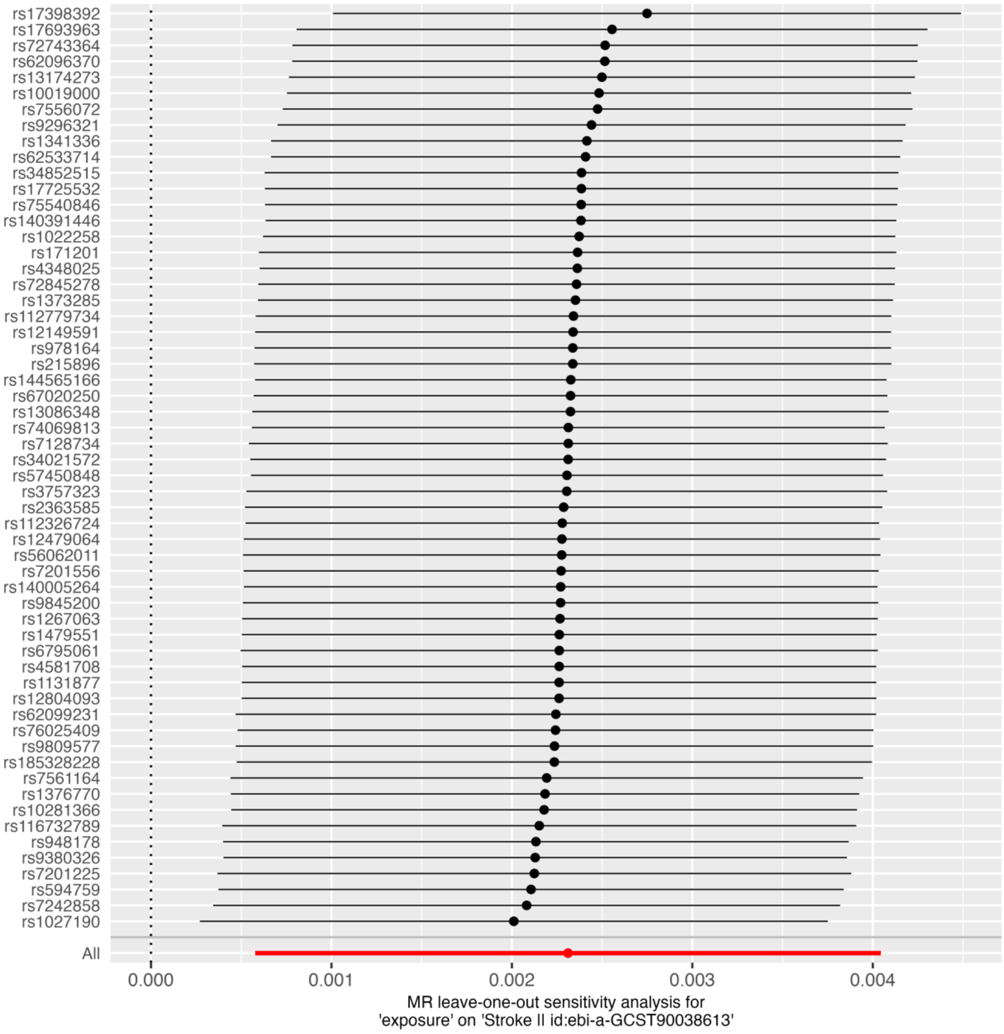

Figure S35. MR sensitivity analysis ($p < 10^{-6}$) of major depressive disorder on stroke. (c) Leave-one-out analysis to assess the influence of single SNPs on overall estimates.

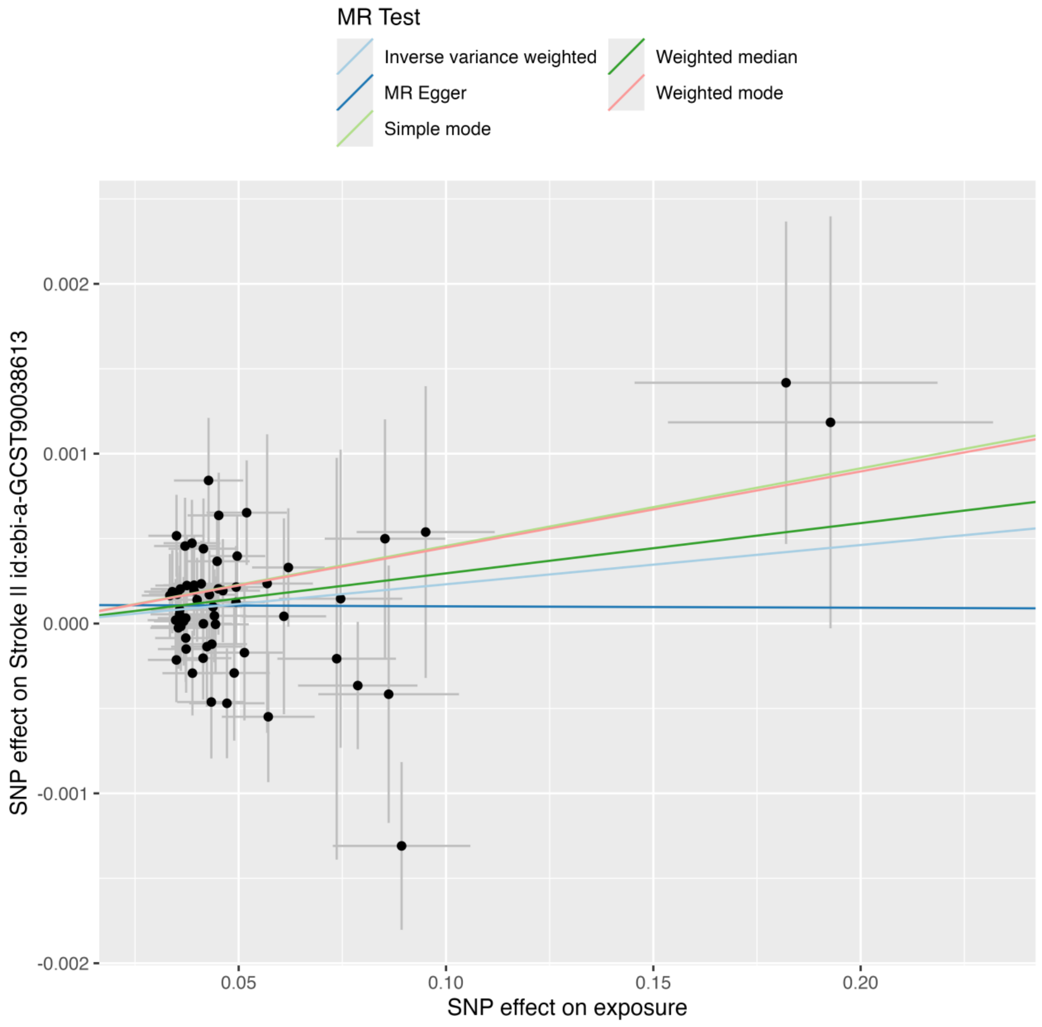

Figure S36. MR sensitivity analysis ($p < 10^{-6}$) of major depressive disorder on stroke. (d) Scatter plot of SNP effects on exposure and outcome with MR regression lines.

Supplementary Tables

Table S1. GWAS sources and characteristics used in the Mendelian randomization analyses

Phenotype and source ID	Definition and effect scale	Cases/controls or N	Ancestry	Build	GWAS adjustment/model	Consortium/source	Potential overlap
Weekly call duration; ukb-b-17999	UKB Field 1120; self-reported call time in the previous 3 months; continuous	N=386,626	European	GRCh37	Age, sex, genotyping array, and genetic principal components in the source UKB GWAS pipeline	UK Biobank/IEU OpenGWAS, 2018	UKB source; overlaps the cohort and UKB-derived outcome GWAS
Depression proxy; finngen_R11_ F5_DEPRESSIO	FinnGen ICD-based depression endpoint; binary log-odds; used as the MDD proxy	53,313/394,756; N=448,069	Finnish	GRCh38	SAIGE with age, sex, genetic principal components, and genotyping batch	FinnGen release 11	No direct UKB overlap; overlap with contributing CVD cohorts cannot be completely excluded
HF; ebi-a-GCST009541	Clinically diagnosed heart failure; binary log-odds	47,309/930,014; N=977,323	European	GRCh37	Study-specific covariates followed by inverse-variance meta-analysis	HERMES	Includes UKB and Finnish contributing studies; exact overlap unavailable
CAD; ebi-a-GCST005195	Coronary artery disease; binary log-odds	122,733/424,528; N=547,261	Mixed; predominantly European	GRCh37	Study-specific covariates followed by consortium meta-analysis	CARDIoGRAMplus C4D and UKB	Includes UKB; possible overlap with the exposure GWAS and cohort
MI; ebi-a-GCST90038610	Myocardial infarction; binary log-odds	11,081/473,517; N=484,598	UKB; catalog ancestry category not reported	GRCh37	Age, sex, genotyping array, and genetic principal components in the source UKB analysis	UK Biobank/GWAS Catalog	Same UKB sampling frame as the exposure GWAS and cohort
Stroke; ebi-a-GCST90038613	Stroke; binary log-odds	6,925/477,673; N=484,598	UKB; catalog ancestry category not reported	GRCh37	Age, sex, genotyping array, and genetic principal components in the source UKB analysis	UK Biobank/GWAS Catalog	Same UKB sampling frame as the exposure GWAS and cohort

Table S2. Primary MR estimates across five estimators using genome-wide significant instruments

Exposure	Outcome	Method	SNPs	OR (95% CI)	P value
Weekly mobile phone call duration	Major depressive disorder	Inverse variance weighted	11	1.559 (1.044–2.328)	0.030
Weekly mobile phone call duration	Major depressive disorder	Weighted median	11	1.356 (0.928–1.981)	0.115
Weekly mobile phone call duration	Major depressive disorder	MR Egger	11	0.365 (0.028–4.674)	0.458
Weekly mobile phone call duration	Major depressive disorder	Simple mode	11	1.310 (0.625–2.747)	0.491
Weekly mobile phone call duration	Major depressive disorder	Weighted mode	11	1.214 (0.625–2.355)	0.580
Weekly mobile phone call duration	Heart failure	Inverse variance weighted	9	1.534 (0.995–2.365)	0.053
Weekly mobile phone call duration	Heart failure	Weighted median	9	1.422 (0.929–2.175)	0.105
Weekly mobile phone call duration	Heart failure	MR Egger	9	4.284 (0.317–57.813)	0.309
Weekly mobile phone call duration	Heart failure	Simple mode	9	1.249 (0.526–2.965)	0.627
Weekly mobile phone call duration	Heart failure	Weighted mode	9	1.239 (0.534–2.872)	0.631
Weekly mobile phone call duration	Coronary artery disease	Inverse variance weighted	9	1.695 (1.127–2.550)	0.011
Weekly mobile phone call duration	Coronary artery disease	Weighted median	9	1.633 (1.111–2.400)	0.013
Weekly mobile phone call duration	Coronary artery disease	MR Egger	9	1.004 (0.089–11.307)	0.998
Weekly mobile phone call duration	Coronary artery disease	Simple mode	9	2.731 (1.177–6.333)	0.047
Weekly mobile phone call duration	Coronary artery disease	Weighted mode	9	2.711 (1.052–6.988)	0.073
Weekly mobile phone call duration	Stroke	Inverse variance weighted	11	1.006 (0.997–1.014)	0.179
Weekly mobile phone call duration	Stroke	Weighted median	11	1.001 (0.990–1.012)	0.905
Weekly mobile phone call duration	Stroke	MR Egger	11	0.950 (0.905–0.997)	0.066
Weekly mobile phone call duration	Stroke	Simple mode	11	0.999 (0.982–1.017)	0.934
Weekly mobile phone call duration	Stroke	Weighted mode	11	0.999 (0.982–1.016)	0.890
Weekly mobile phone call duration	Myocardial infarction	Inverse variance weighted	11	1.010 (0.999–1.021)	0.072
Weekly mobile phone call duration	Myocardial infarction	Weighted median	11	1.012 (0.998–1.027)	0.097
Weekly mobile phone call duration	Myocardial infarction	MR Egger	11	0.965 (0.908–1.026)	0.285
Weekly mobile phone call duration	Myocardial infarction	Simple mode	11	1.017 (0.993–1.041)	0.195
Weekly mobile phone call duration	Myocardial infarction	Weighted mode	11	1.017 (0.990–1.044)	0.255
Major depressive disorder	Heart failure	Inverse variance weighted	20	1.087 (0.990–1.194)	0.080
Major depressive disorder	Heart failure	Weighted median	20	1.072 (0.944–1.218)	0.285
Major depressive disorder	Heart failure	MR Egger	20	1.089 (0.638–1.860)	0.758
Major depressive disorder	Heart failure	Simple mode	20	1.102 (0.890–1.365)	0.385
Major depressive disorder	Heart failure	Weighted mode	20	1.107 (0.902–1.358)	0.344
Major depressive disorder	Coronary artery disease	Inverse variance weighted	19	1.203 (1.089–1.328)	< 0.001
Major depressive disorder	Coronary artery disease	Weighted median	19	1.166 (1.036–1.313)	0.011
Major depressive disorder	Coronary artery disease	MR Egger	19	1.126 (0.655–1.936)	0.672
Major depressive disorder	Coronary artery disease	Simple mode	19	1.177 (0.970–1.427)	0.116
Major depressive disorder	Coronary artery disease	Weighted mode	19	1.177 (0.985–1.405)	0.089
Major depressive disorder	Stroke	Inverse variance weighted	24	1.003 (1.001–1.005)	0.018
Major depressive disorder	Stroke	Weighted median	24	1.003 (1.000–1.007)	0.062
Major depressive disorder	Stroke	MR Egger	24	1.000 (0.987–1.013)	0.999
Major depressive disorder	Stroke	Simple mode	24	1.004 (0.998–1.010)	0.220
Major depressive disorder	Stroke	Weighted mode	24	1.004 (0.997–1.010)	0.256
Major depressive disorder	Myocardial infarction	Inverse variance weighted	24	1.004 (1.001–1.007)	0.008
Major depressive disorder	Myocardial infarction	Weighted median	24	1.004 (1.000–1.009)	0.059
Major depressive disorder	Myocardial infarction	MR Egger	24	1.006 (0.990–1.023)	0.456
Major depressive disorder	Myocardial infarction	Simple mode	24	0.999 (0.990–1.007)	0.740
Major depressive disorder	Myocardial infarction	Weighted mode	24	1.007 (0.998–1.015)	0.146

Table S3. Instrument-strength, heterogeneity, and pleiotropy diagnostics for the primary and sensitivity-threshold MR analyses

Exposure–outcome	Threshold	SNPs	Minimum F	Mean F	Median F	Cochran's Q	Q df	Q P	I^2 (%)	Egger intercept (P)
Call duration–MDD	$p < 5 \times 10^{-8}$	11	31.20	37.12	34.33	32.51	10	< 0.001	69.2	0.0296 (0.288)
Call duration–HF	$p < 5 \times 10^{-8}$	9	31.20	37.88	34.33	18.61	8	0.017	57.0	-0.0212 (0.458)
Call duration–CAD	$p < 5 \times 10^{-8}$	9	31.20	37.88	34.33	21.02	8	0.007	61.9	0.0109 (0.679)
Call duration–STROKE	$p < 5 \times 10^{-8}$	11	31.20	37.12	34.33	10.10	10	0.432	0.9	0.0012 (0.043)
Call duration–MI	$p < 5 \times 10^{-8}$	11	31.20	37.12	34.33	11.49	10	0.321	13.0	0.0009 (0.175)
MDD–HF	$p < 5 \times 10^{-8}$	20	29.92	37.29	35.13	20.27	19	0.378	6.3	-0.0001 (0.995)
MDD–CAD	$p < 5 \times 10^{-8}$	19	29.92	36.61	34.45	27.31	18	0.073	34.1	0.0031 (0.812)
MDD–STROKE	$p < 5 \times 10^{-8}$	24	29.92	36.83	34.02	16.49	23	0.833	0.0	0.0001 (0.653)
MDD–MI	$p < 5 \times 10^{-8}$	24	29.92	36.83	34.02	21.43	23	0.555	0.0	-0.0001 (0.793)
Call duration–MDD	$p < 10^{-6}$	39	24.09	29.42	28.15	77.12	38	< 0.001	50.7	0.0008 (0.925)
Call duration–HF	$p < 10^{-6}$	32	24.09	29.58	27.84	46.15	31	0.039	32.8	-0.0025 (0.752)
Call duration–CAD	$p < 10^{-6}$	32	24.09	29.58	27.84	51.00	31	0.013	39.2	0.0023 (0.758)
Call duration–STROKE	$p < 10^{-6}$	40	24.09	29.42	28.21	26.77	39	0.931	0.0	0.0002 (0.189)
Call duration–MI	$p < 10^{-6}$	40	24.09	29.42	28.21	54.84	39	0.048	28.9	0.0002 (0.373)
MDD–HF	$p < 10^{-6}$	53	24.06	30.37	27.02	53.27	52	0.425	2.4	0.0054 (0.271)
MDD–CAD	$p < 10^{-6}$	52	24.06	29.96	26.91	82.18	51	0.004	37.9	0.0065 (0.268)
MDD–STROKE	$p < 10^{-6}$	58	24.06	30.56	27.61	58.01	57	0.438	1.7	0.0001 (0.460)
MDD–MI	$p < 10^{-6}$	58	24.06	30.56	27.61	75.94	57	0.048	24.9	0.0001 (0.713)

F statistics summarize the instrument–exposure associations; F_{min}, mean F, and median F are reported for each threshold. Cochran's Q and I^2 are reported for the inverse-variance weighted model. The MR-Egger intercept tests directional horizontal pleiotropy.

Table S4. Scaled Schoenfeld-residual tests for the fully adjusted Cox models

Outcome	Phone-use χ^2	df	Phone-use P	Global χ^2 (df)	Global P
HF	7.84	5	0.165	61.63 (23)	< 0.001
CAD	9.68	5	0.085	189.81 (23)	< 0.001
Stroke	13.24	5	0.021	73.69 (23)	< 0.001
MI	3.87	5	0.569	101.70 (23)	< 0.001

The phone-use test jointly assesses the five non-reference call-duration categories. A significant global test indicates non-proportionality for at least one term in the full model; it does not by itself imply non-proportionality of the phone-use term.

Table S5. Fully adjusted Cox estimates stratified by baseline MDD status for CAD

Weekly call duration	Baseline MDD HR (95% CI)	P value	No baseline MDD HR (95% CI)	P value
<5 min	Reference		Reference	
5-29 min	1.97 (0.87–1.09)	0.648	1.04 (1.00–1.08)	0.034
30-59 min	0.96 (0.84–1.10)	0.566	1.04 (1.00–1.09)	0.069
1-3 h	1.08 (0.93–1.24)	0.320	1.11 (1.06–1.16)	< 0.001
4-6 h	1.07 (0.85–1.35)	0.561	1.14 (1.06–1.23)	< 0.001
>6 h	1.25 (1.00–1.57)	0.046	1.18 (1.09–1.27)	< 0.001

Baseline MDD: 2191 events/26015 participants; no baseline MDD: 22032 events/294328 participants. Model 4 adjusted for age, sex, BMI, Townsend deprivation index, diabetes, cancer, smoking, alcohol use, sleep pattern, and education. The 5-df global interaction P value was 0.591; stratum-specific estimates are descriptive.

Table S6. Fully adjusted Cox estimates stratified by baseline MDD status for HF

Weekly call duration	Baseline MDD HR (95% CI)	P value	No baseline MDD HR (95% CI)	P value
<5 min	Reference		Reference	
5-29 min	1.06 (0.90–1.25)	0.497	1.00 (0.95–1.05)	0.950
30-59 min	1.11 (0.91–1.35)	0.324	1.01 (0.94–1.08)	0.817
1-3 h	1.20 (0.97–1.49)	0.091	1.01 (0.94–1.09)	0.797
4-6 h	1.17 (0.83–1.66)	0.369	0.93 (0.82–1.05)	0.229
>6 h	1.41 (1.01–1.97)	0.043	1.02 (0.90–1.15)	0.808

Baseline MDD: 1021 events/26803 participants; no baseline MDD: 9372 events/301367 participants. Model 4 adjusted for age, sex, BMI, Townsend deprivation index, diabetes, cancer, smoking, alcohol use, sleep pattern, and education. The 5-df global interaction P value was 0.115; stratum-specific estimates are descriptive.

Table S7. Fully adjusted Cox estimates stratified by baseline MDD status for MI

Weekly call duration	Baseline MDD HR (95% CI)	P value	No baseline MDD HR (95% CI)	P value
<5 min	Reference		Reference	
5-29 min	1.08 (0.88–1.33)	0.449	1.03 (0.97–1.09)	0.325
30-59 min	1.04 (0.81–1.33)	0.748	1.00 (0.93–1.08)	0.981
1-3 h	1.17 (0.90–1.51)	0.237	1.05 (0.97–1.14)	0.197
4-6 h	1.02 (0.67–1.55)	0.934	1.01 (0.89–1.15)	0.878
>6 h	0.87 (0.55–1.36)	0.528	1.10 (0.97–1.24)	0.127

Baseline MDD: 693 events/26323 participants; no baseline MDD: 7545 events/296521 participants. Model 4 adjusted for age, sex, BMI, Townsend deprivation index, diabetes, cancer, smoking, alcohol use, sleep pattern, and education. The 5-df global interaction P value was 0.778; stratum-specific estimates are descriptive.

Table S8. Fully adjusted Cox estimates stratified by baseline MDD status for Stroke

Weekly call duration	Baseline MDD HR (95% CI)	P value	No baseline MDD HR (95% CI)	P value
<5 min	Reference		Reference	
5-29 min	1.04 (0.90–1.20)	0.610	1.01 (0.96–1.06)	0.662
30-59 min	0.95 (0.80–1.14)	0.611	1.03 (0.97–1.09)	0.291
1-3 h	0.96 (0.78–1.17)	0.673	1.12 (1.05–1.19)	< 0.001
4-6 h	1.24 (0.92–1.68)	0.157	1.10 (0.99–1.23)	0.079
>6 h	1.11 (0.80–1.53)	0.531	1.18 (1.05–1.31)	0.004

Baseline MDD: 1241 events/26322 participants; no baseline MDD: 11628 events/298293 participants. Model 4 adjusted for age, sex, BMI, Townsend deprivation index, diabetes, cancer, smoking, alcohol use, sleep pattern, and education. The 5-df global interaction P value was 0.519; stratum-specific estimates are descriptive.

Table S9. CVDs and corresponding ICD-10 codes used in this study from UK Biobank.

Outcomes	UKB Field ID	Field names	Data codes
Coronary artery disease	41270 40001 40002	Diagnoses - ICD10 Underlying (primary) cause of death: ICD10 Contributory (secondary) cause of death: ICD10	I21, I210, I211, I212, I213, I214, I219, I21X, I22, I220, I221, I228, I229, I23, I230, I231, I232, I233, I234, I235, I236, I238, I24, I240, I241, I248, I249, I25, I250, I251, I252, I253, I254, I255, I256, I258, I259
Heart failure	41270 40001 40002	Diagnoses - ICD10 Underlying (primary) cause of death: ICD10 Contributory (secondary) cause of death: ICD10	I500, I501, I509
Stroke	41270 40001 40002	Diagnoses - ICD10 Underlying (primary) cause of death: ICD10 Contributory (secondary) cause of death: ICD10	I60, I600, I601, I602, I603, I604, I605, I606, I607, I608, I609, I61, I610, I611, I612, I613, I614, I615, I616, I618, I619, I62, I620, I621, I629, I63, I630, I631, I632, I633, I634, I635, I636, I638, I639, I64
Myocardial infarction	41270 40001 40002	Diagnoses - ICD10 Underlying (primary) cause of death: ICD10 Contributory (secondary) cause of death: ICD10	I21, I210, I211, I212, I213, I214, I219, I22, I220, I221, I228, I229

Table S10. MDD and corresponding ICD-10 codes used in this study from UK Biobank.

Outcomes	UKB Field ID	Field names	Data codes
Major depressive disorder	41270 40001 40002	Diagnoses - ICD10 Underlying (primary) cause of death: ICD10 Contributory (secondary) cause of death: ICD10	F32, F320, F321, F322, F323, F328, F329, F33, F330, F331, F332, F333, F334, F338, F339

Table S11. List of risk factors and their UK Biobank codes used in this study.

Field ID	Description
21001	Body mass index (BMI)
21022	Age
31	Gender
22189	Townsend deprivation index
20117	Drinking status
20116	Smoking status
6138	Education
1120	Weekly usage of mobile phone in last 3 months
1160	Sleep duration
1180	Morning/evening person(chronotype)
1200	Insomnia
1210	Snoring
1220	Daytime dozing
2443	Diabetes
2453	Cancer
50	Height
